# MicrobeRX: a tool for enzymatic-reaction-based metabolite prediction in the gut microbiome

**DOI:** 10.1186/s40168-025-02070-5

**Published:** 2025-03-19

**Authors:** Angel J. Ruiz-Moreno, Ángela Del Castillo-Izquierdo, Isabel Tamargo-Rubio, Jingyuan Fu

**Affiliations:** 1 Department of Genetics, University Medical Center Groningen, Groningen, 9713GZ The Netherlands; 2 Department of Pediatrics, University Medical Center Groningen, Groningen, 9713GZ The Netherlands; 3 Department of Medical Microbiology, University Medical Center Groningen, Groningen, 9713GZ The Netherlands

## Abstract

**Background:**

The gut microbiome functions as a metabolic organ, producing numerous enzymes that influence host health; however, their substrates and metabolites remain largely unknown.

**Results:**

We present MicrobeRX, an enzyme-based metabolite prediction tool that employs 5487 human reactions and 4030 unique microbial reactions from 6286 genome-scale models, as well as 3650 drug metabolic reactions from the DrugBank database (v.5.1.12). MicrobeRX includes additional analysis modules for metabolite visualization and enzymatic and taxonomic analyses. When we applied MicrobeRX to 1083 orally administered drugs that have been approved in at least one jurisdiction at some point in time (DrugBank), it predicted metabolites with physicochemical properties and structures similar to metabolites found in biosamples (from MiMeDB). It also outperformed another existing metabolite prediction tool (BioTransformer 3.0) in terms of predictive potential, molecular diversity, reduction of redundant predictions, and enzyme annotation.

**Conclusions:**

Our analysis revealed both unique and overlapping metabolic capabilities in human and microbial metabolism and chemo- and taxa-specific microbial biotransformations. MicrobeRX bridges the genomic and chemical spaces of the gut microbiome, making it a valuable tool for unlocking the chemical potential of the gut microbiome in human health, the food and pharmaceutical industries, and environmental safety.

Video Abstract

**Supplementary Information:**

The online version contains supplementary material available at 10.1186/s40168-025-02070-5.

## Introduction

The importance of the gut microbiome in human health and disease has become increasingly recognized due to its pivotal role in physiological processes such as metabolism [1–3] and immune system modulation [4, 5], as well as in other functions [6, 7]. The gut microbiome’s capacity to metabolize dietary compounds and xenobiotics [1] is particularly relevant because it can directly affect nutrition [8], drug response [9], and disease outcomes [9–15]. However, as the majority of the metabolites produced by the gut microbiome remain unknown, our understanding of their effects on human health remains limited [10, 16].

To date, association analysis using human cohorts has emerged as a powerful tool for establishing associations between gut microbes and metabolites [11, 13], but this approach has many limitations. One is related to current metabolic profiling methods such as liquid chromatography–mass spectrometry, gas chromatography–mass spectrometry, and nuclear magnetic resonance spectrometry, all of which can only detect certain types of metabolites, depending on their technical settings. While untargeted approaches can identify millions of mass peaks, only around 1200–1500 metabolites can be annotated, and qualitative and quantitative identification of most metabolites remains difficult [17–19]. Another limitation is that the metabolite levels measured in fecal and plasma samples are the result of complex interactions between the host environment (including diet), genetics, and gut microbiome. Variations in their plasma concentrations are linked to a variety of biological processes such as metabolic uptake, biotransformation, transportation, and secretion [20, 21], making it difficult to establish a direct mechanistic link between metabolite abundance and microbial enzyme function. Culturomics-based in vitro experiments are a valuable method for studying the enzymatic capacity of gut microbes, but these experiments are often low throughput and designed to address a specific hypothesis. Furthermore, these approaches have failed to provide a comprehensive understanding of the vast chemical structures of microbial metabolites [22, 23]. At present, the diversity and complexity of this biosystem means that obtaining a complete picture of the microbial metabolism of chemicals remains a significant challenge [2, 7].

To address this challenge, there is increasing interest in using genomic and cheminformatic approaches to predict the bacterial metabolism of biotic and xenobiotic molecules, with established predictive tools falling broadly into three categories. One category focuses on the genomic annotation and prediction of bacterial enzymes, based on substrates. GutBug, for example, can predict the enzyme commission (EC) number of bacterial enzymes and the gut microbes harboring these enzymes, based on specific chemical substrates [24]. SIMMER uses similarity-based algorithms to predict species and enzymes capable of known chemical transformations, with MetaCyc serving as the source for biochemical transformations [25]. Similarly, MicrobeFDT groups chemically similar drug and food compounds and links them to microbial enzymes and known toxicities [26]. However, these tools do not predict metabolic products. The second category of tools predicts metabolites using bacterial enzymes and pathways. Examples here include PRISM 4, which uses bacterial genome biosynthesis gene clusters to predict metabolic products and their chemical structures [27]. The third category predicts metabolic profiles (the type and abundance of metabolites) based on metagenomics, and examples include ENVIM [28] and MMINP [29]. However, all these existing methods rely on previously defined substrate–enzyme–product relationships, often with only one substrate described for a single chemical reaction, which limits their potential to discover novel metabolites. Thus, most substrates and metabolic products of gut microbial metabolism remain “dark matter.”

To our knowledge, BioTransformer 3.0 [30, 31] is the only publicly available tool that can chemically predict novel metabolites from both humans and the gut microbiome. However, phase I metabolism from BioTransformer primarily focuses on human CYP450 transformations. For microbial transformation, BioTransformer does not provide detailed descriptions of the enzymes and microbial organisms involved, which significantly reduces its utility for comprehensive analysis and comparison of human and gut microbial metabolism. Therefore, we developed MicrobeRX, a novel knowledge-based tool that uses data from genome-scale metabolic models (GEMs) [32] to comprehensively predict the chemical structures of metabolites in the metabolic space of both humans and the human gut microbiome. MicrobeRX integrates biotransformation reactions from a human GEM from Recon3D [33] and 6286 microbial strain–resolved GEMs derived from AGORA2 [32]. MicrobeRX is uniquely equipped with multiple analysis modules and various sources of information to score, analyze, and investigate physicochemical properties, organism information, enzyme annotations, and the metabolic pathways of the predicted metabolites.

To demonstrate its application in the discovery of novel metabolites, we applied MicrobeRX to all 1083 approved orally administered drugs in DrugBank [34]. This identified 7020 structurally diverse drug metabolites resulting from human biotransformation, 5878 from biotransformation by microbial enzymes, and 734 metabolites produced by both. Comparative cheminformatics analysis revealed human- and microbiome-specific biotransformations, as well as host–microbe co-metabolism. To benchmark the predictions, we compared them to real metabolites from the Microbial Metabolites Database (MiMeDB) [35] and to results from BioTransformer 3.0.

## Results

### Development of microbeRX

**Fig. 1:**
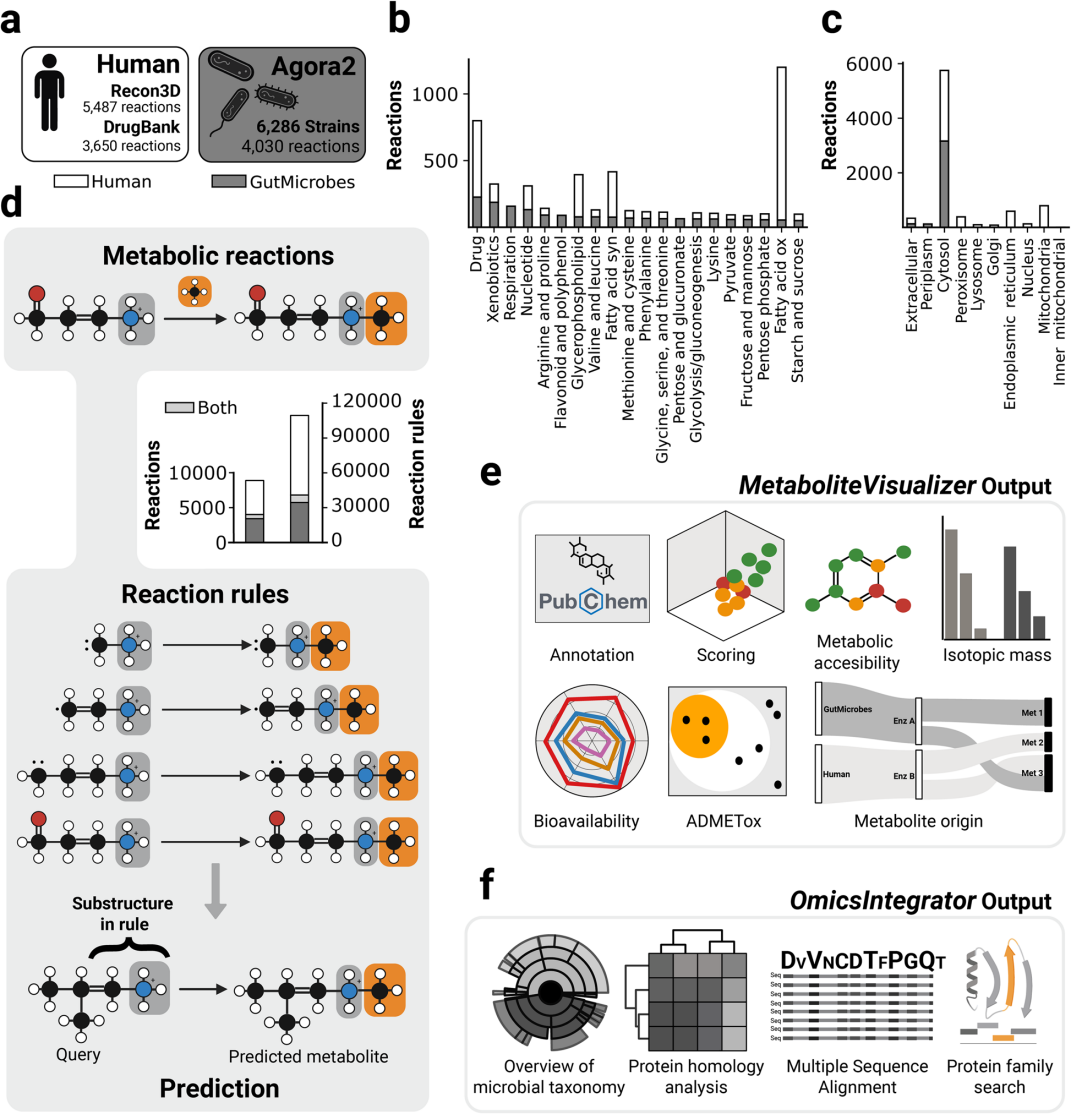
Data from genome-scale metabolic models (GEMs) and modules are available in MicrobeRX.** a** Schematic representation of the sources of the metabolic reactions is included in this project. **b** Top 20 metabolic pathways (gray: gut microbes, white: human Recon3D). **c** Cellular localization of the metabolic reactions obtained from humans (white bar, Recon3D) and gut microbes (gray bar, 6286 strain-resolved GEMs from AGORA2). **d** Decomposition of the metabolic reaction into chemically specific reaction rules (CSRRs) with varying atom numbers. The central bar plot shows the CSRRs produced per biosystem (dark gray: microbes, white: human, light gray: both). The reactive atom in the metabolic reaction scheme is highlighted with a gray box (carbon: black, nitrogen: blue, oxygen: red, hydrogen: white). The structure added (methane for example) is highlighted with an orange box. The brace in the prediction scheme indicates the substructure shared by the query and the substrate in the CSRR (three heteroatoms “C = C–N”). **e** Plots available in the *MetaboliteVisualizer* module include PubChem annotation, 3D scoring plot, metabolic accessibility diagram, isotopic mass decomposition, bioavailability (Lipinski’s rule of five), ADMETox (BOILED-Egg), and Sankey for relationships between origin, enzyme, and metabolite. See Fig. S6 for examples of real plots from *MetaboliteVisualizer*. **f** Plots available in the *OmicsIntegrator* module include sunburst of microorganisms per reaction/enzyme, cluster map of pairwise protein sequence comparisons for homology analysis, multiple sequence alignment with the consensus sequence (protein logo), and protein family search in InterPro. See Fig. S7 for examples of real plots from *OmicsIntegrator*

The data sources and analysis framework of MicrobeRX are presented in Fig. 1. We first obtained up-to-date GEMs from both human and human gut microbiomes, including 13,540 reactions from a human GEM derived from Recon3D [33] and 8638 reactions from 6286 strain-resolved GEMs derived from AGORA2 [32] (Supplementary Table 1). These 6286 strains could be classified into 1396 species. We then assessed how well these species represent the human gut microbiome by assessing their collective abundance in 8482 fecal metagenomics samples from the Dutch Microbiome Project [36]. We were able to identify abundances for 330 species, collectively accounting for 77% of total gut microbial composition on average (Fig. S1).

As MicrobeRX focuses on biotransformation, we filtered out reactions involving transport, exchange, stereochemical conversions, passive diffusion, and biomass production. This left 5487 human metabolic reactions and 4030 gut microbial metabolic reactions, resulting in 8905 unique reactions (Fig. 1a). Of these, 4875 reactions are human-specific, 3418 are microbiome-specific, and 612 are shared between human and gut microbiome. Comparing the biological processes of these reactions, we noticed that a large number of human reactions participate in fatty acid synthesis and oxidation, while drug- and xenobiotic-related reactions are among the top categories for both human and gut microbiome (Fig. 1b). Cellular compartment analysis showed that the metabolic reactions in human and gut microbiome were mainly conducted in the cytosolic compartment (Fig. 1c). To improve the traceability of reactions across GEMs and various metabolic databases, we used MetaNetX V4 [37] to cross-reference all substrates and biochemical reactions and integrated data from all well-known public biochemical databases and GEMs, including RHEA [38], MetaCyc [39], KEGG [40], PubSEED [41], and BiGG [42].

To enable the prediction of novel metabolites with similar key chemical features [43, 44], we generalized the 8905 unique reactions to 109,403 Chemically Specific Reaction Rules (CSRRs). A CSRR is a simplified representation of a chemical reaction that retains detailed information regarding key atom changes and bond formation or breakage (Fig. 1d). As a result, this method identifies the functional groups and chemical patterns required for a chemical reaction to occur. CSRR are also a very versatile format for reproducing chemical reactions. Multiple CSRRs can be generated for one reaction depending on the number of heteroatoms included in the CSRRs. To facilitate CSRR generation, we developed the *RuleGenerator* module, which generates CSRRs from genome-derived GEMs and databases (see Materials and Methods, Fig. S2). Using *RuleGenerator* in Recon3D and AGORA2 GEMs, we generated 68,511 CSRRs for 4875 human-specific reactions (average of 14 CSRRs per reaction) and 34,478 CSRRs for 3418 microbiome-specific reactions (average of 10 CSRRs per reaction). To further facilitate drug metabolite prediction, MicrobeRX also incorporates expanded reaction rules and includes 19,560 CSRRs derived from 3650 drug metabolic reactions from the DrugBank v.5.1.12 (Supplementary Table 2). When more heteroatoms are included in a CSRR, it becomes more specific and accurate but also has less potential for discovering novel compounds. The fewer heteroatoms included, the simpler the CSRR and the greater the likelihood of producing false positive predictions. To address this issue, we developed a confidence score metric based on the structural similarity of the predicted metabolites to the real substrates and products in the reaction (Fig. S3). We also included an atom efficiency metric in our scoring system that represents the ratio of heteroatoms in the query to heteroatoms in the query–CSRR common substructure.

MicrobeRX also includes multiple integrative analysis and visualization modules for the downstream analysis of predicted metabolites, enzymes, and organisms (Fig. 1e). The *MetaboliteAnalyzer* module integrates PubChem [45] to search for annotations of metabolites, PyOpenMS [46] to compute the isotopic mass decomposition, RDKit (www.rdkit.org) to compute molecular descriptors, and ClassyFire [47] to classify the metabolites based on their structural features. We also created the *MetaboliteVisualizer* module, which generates interactive plots based on the analysis above and the confidence score. For example, the visualizer is used to generate Lipinski’s rule of five and Brain Or IntestinaL EstimateD permeation [48] (BOILED-Egg) plots, which aid in the study of the bioavailability and ADMETox properties of the metabolites. *MetaboliteAnalyzer* can also generate a metabolic accessibility diagram that shows which chemical substructures of the query are more likely to be metabolized [49, 50] (Fig. S4). Finally, this module uses extensive data from the results table to plot complex relationships such as the origin, the enzyme involved in the transformation, and the predicted metabolite.

One of the primary goals of MicrobeRX is to characterize the predicted metabolites in the context of the enzymes and organisms involved in the biotransformations. For this we developed the *OmicsIntegrator* module, which provides a more in-depth genomic analysis of the enzymes and organisms (Fig. 1f). It facilitates homology analysis and identification of common sequence patterns in various organisms through pairwise and multiple sequence alignment (MSA). This module also enables the investigation of relevant protein families and domains using InterPro [51].

By combining large amounts of data from numerous sources, a novel prediction pipeline, and comprehensive downstream metabolic, genomic, and structural analysis modules, our tool can provide insight into how enzymes transform small molecules. MicrobeRX is a Python-based tool (Fig. S**5**) that can be integrated with other chemical and bioinformatics tools. It is freely available at https://pypi.org/project/MicrobeRX/, with documentation available at https://microberx.readthedocs.io/en/latest/.

Altogether, MicrobeRX provides extensive information about the predictions and their biochemical context, e.g., reaction cofactors, system or pathway, origin, confidence, and cross-references. An example of the prediction outputs for hydrocortisone (a treatment for endocrine disorders, immune conditions, and allergic disorders) is displayed in Supplementary Table 3 and Figs. S6 and **S7**.

#### Benchmarking with DrugBank drug metabolites

To demonstrate MicrobeRX’s predictive ability, we benchmarked its performance using DrugBank drug metabolites and then compared its performance against BioTransformer. DrugBank v.5.1.12 stores information for 11,928 drug structures, as well as both primary and secondary drug metabolites. However, chemical reaction information is only available for 3,650 reactions (Supplementary Table 2) involving 954 drugs, with 1–20 drug metabolites reported per drug (Fig. S8a,b). Most of the enzymes reported are members of the CYP family and UDP-glucuronosyltransferases (Fig. S8c).

We first assessed to what extent MicrobeRX can correctly predict known drug metabolites. Based on DrugBank CSRRs, MicrobeRX predicted 480,458 drug metabolites for the 954 drugs. When we examined the distribution of the confidence scores of our predictions, the top whisker (Q3 + 1.5*IQR) was 1.206. We then regarded the 14,058 predicted metabolites that exceeded this threshold as confident predictions (Fig. S8d). Next, we identified correct predictions where the predicted metabolites and the DrugBank CSRR exactly matched the reported metabolite and metabolic reaction. In all, MicrobeRX correctly predicted 63.2% (2307) of the primary and secondary metabolites (Fig. S8**e**).

We note that MicrobeRX could not predict 37% of metabolites. This is due to scenarios in which the DrugBank reactions do not meet the CSRR requirements, primarily because of incomplete reaction information. DrugBank focuses on reporting the direct metabolite for each drug, leaving out other agents of chemical reactions such as cofactors. This significantly influences MicrobeRX’s ability to produce the corresponding metabolites because incomplete atom-mapping results in invalid chemical transformations. Additionally, some of the reported metabolites come from large, complex reactions that are difficult to simplify. For example, for metabolic conversions that are achieved through a series of enzymatic steps, but where the reported reaction only includes the initial substrate and final product, it is not possible to determine an adequately representative CSRR.

**Fig. 2:**
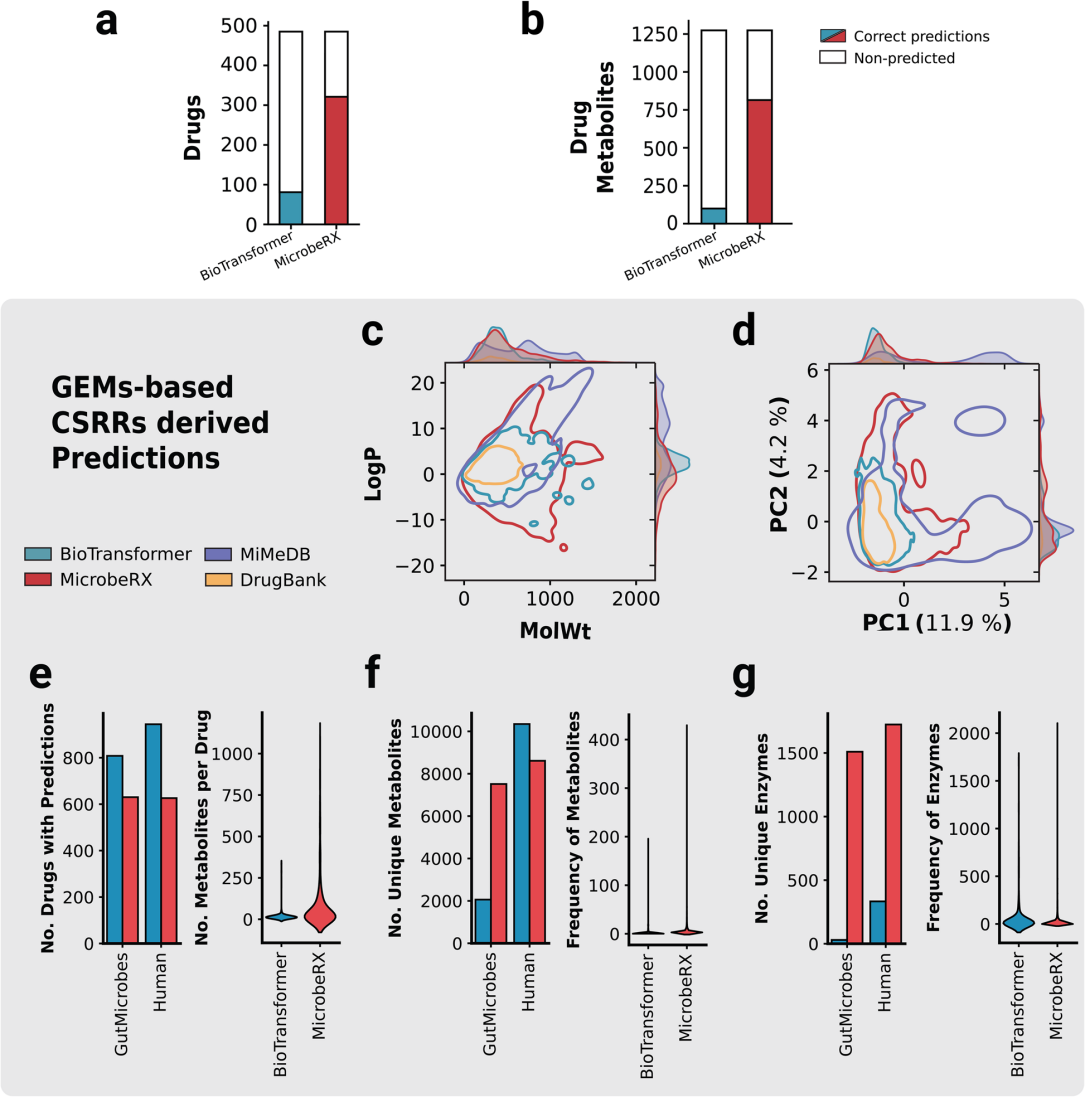
Benchmark and comparison of MicrobeRX with BioTransformer, DrugBank metabolites, and MiMeDB.** a** Number of orally administered drugs (485) correctly predicted by BioTransformer (81) and MicrobeRX (321). **b** Number of drug metabolites (1274) correctly predicted by BioTransformer (98) and by MicrobeRX (812). **c** Molecular weight vs. LogP comparison for predicted metabolites and DrugBank and MiMeDB metabolites. **d** Principal component analysis (PCA) of molecular similarity between the predictive tools and metabolite datasets using Morgan fingerprints (2048 bits, radius ≥ 2). Duplicated molecules were removed from each dataset prior to comparison. **e** Number of drugs predicted to be metabolized by microbial or human reactions (barplot at left) and number of metabolites per drug produced per tool (violin plot at right). **f** Number of unique metabolic structures produced for microbial and human reactions (barplot at left) and the frequency at which each metabolite is predicted (violin plot at right). **g** Number of unique microbial and human enzyme annotations used for the predictions (barplot at left) and the frequency at which each enzyme is found in the predictions (violin plot at right). In the bar plots in panels **e–g**, the red bar indicates MicrobeRX predictions and the blue bar indicates BioTransformer predictions

Next, we compared MicrobeRX’s predictive potential with that of BioTransformer. However, as BioTransformer requires the use of specific sequential modules per input to predict the primary and secondary metabolites, we restricted this comparison to one-step metabolites of orally administered drugs, which involved 485 drugs and 1274 drug metabolites (1144 diverse chemical compounds). Since BioTransformer has no scoring metric to assess the reliability of predictions, we used all of its predictions for comparison. Furthermore, BioTransformer provides generic names for enzymatic reactions, e.g., several members of the CYP family are classified into one reaction named “Hydroxylation of terminal methyl.” We therefore considered BioTransformer predictions as correct only when the structure of the compound produced was found in the drug metabolites dataset. In all, BioTransformer predicted 6337 metabolites, correctly predicting 16.70% (81) of the drugs and 7.70% (98) of the metabolites. By contrast, MicrobeRX correctly predicted 66.01% (321) of the drugs (Fig. 2a) and 63.7% (812) of the metabolites (Fig. 2b). In addition, 61.22% (60) of the BioTransformer predictions were also found in MicrobeRX (Supplementary Table 2). One notable reason for the differences in prediction performance is that MicrobeRX has included DrugBank reaction rules, while such reaction information may be missing in Biotransformer. This highlights a general limitation of such prediction tools: no good reaction information, no good prediction. The *RuleGenerator* module in MicrobeRX allows users to consistently update reaction information.

#### Comparison of predicted metabolites for all orally administered drugs

Encouraged by our tool’s prediction capability, and with the goal of predicting novel metabolites from human and gut microbiome, we proceeded to predict the metabolites for all 1083 approved, orally administered drugs in DrugBank, including those without reported metabolites in the database (Supplementary Table 4). Since the DrugBank metabolite dataset is enriched in human products of unknown enzymatic origin, CYP family members, and UDP-glucuronosyltransferases, to generate unbiased predictions towards these biotransformations, we decided to apply only the GEM-based CSRRs derived from humans (Recon3D) and microbes (AGORA2). MicrobeRX predicted 485,033 metabolic structures for 1074 drugs. Unsurprisingly, CSRRs with fewer atoms predicted more drugs (Fig. S8f) and metabolites (Fig. S8g) and lower confidence scores led to the inclusion of more redundant and possibly false positive predictions (Fig. S8h). Based on the previous selection cutoff, we kept the prediction confidence score cutoff of ≥ 1.2 for high-confidence predictions. This confidence level indicates at least 40% molecular similarity between the query and the real substrate, as well as between the predicted metabolite and the actual product of the biotransformation. After filtering out low-confidence predictions, MicrobeRX generated 53,811 predictions (13,632 unique metabolites) for 731 drugs (Supplementary Table 5). By contrast, BioTransformer generated 15,979 (11,047 unique metabolites) predictions for 1003 drugs (Supplementary Table 6). Only 19 metabolites were predicted by both MicrobeRX and BioTransformer.

Interestingly, MicrobeRX did not produce results for nine drugs with complex fused rings, including the adamantane derivatives amantadine and memantine. The DrugBank metabolism entry for these compounds states “No appreciable metabolism, although negligible amounts of an acetyl metabolite have been identified,” which appears to be consistent with our findings. On the other hand, for the compounds with predictions with a confidence score below 1.2, we did not observe any distinct pattern of molecular similarity when we compared them with the drugs that produced high-confidence predictions. We attribute this behavior to how the specificity of the CSRR enables it to capture diverse functional groups. For example, nilutamide (DB00665) has a nitroaromatic ring that shares structural similarities with other nitroaromatic drugs such as nitrazepam and clonazepam. However, nilutamide has a trifluoromethane group in ortho position with respect to the nitro group, which makes this compound more likely to match with CSRR with fewer numbers of atoms (i.e., less specific) for nitro aromatic reduction, yielding predictions with a lower confidence score.

We further questioned whether these predictions were chemically reasonable and hypothesized that the predictions should be physiochemically and structurally similar to metabolites from biological samples. We therefore compared the features of the predictions to DrugBank metabolites and 24,039 microbial metabolites from the Microbial Metabolites Database (MiMeDB) [35]. Although few metabolite structures from the predictions were found in the metabolites datasets, their physicochemical and structural profiles showed substantial similarity to reported metabolites (Fig. 2c,d, Supplementary Table 7) [52]. Interestingly, DrugBank metabolites seem to comply with Lipinski’s rule of five (molecular weight ≤ 500 Daltons, octanol–water partition coefficient -LogP- ≤ 5, hydrogen bond donors ≤ 5, and hydrogen bond acceptors ≤ 10) [53, 54], whereas MiMeDB metabolites exhibit greater dispersion in terms of LogP, indicating increased lipophilicity and mass [52]. When making the comparison, we discovered that MicrobeRX produces compounds with a wider range of structural diversity that completely covers the DrugBank metabolites dataset, the BioTransformer predictions, and most of the chemical space of the MiMeDB dataset. This was especially striking because the MicrobeRX predictions are based on orally administered drugs, whereas MiMeDB contains xenobiotic metabolites from a variety of environmental sources. This is in line with previous evidence indicating that the chemical spaces of metabolites from human and gut microbiota are limited in their physicochemical properties and structural diversity [52].

To investigate the differences between the predictive capabilities of MicrobeRX and BioTransformer in more detail, we compared their results. While BioTransformer processed more drugs (Fig. 2e), it often reported redundant low-mass secondary products such as dimethylamine, acetaldehyde, carbon dioxide, and methylamine (Supplementary Table 6). This was particularly interesting because, while BioTransformer seems to produce a higher number of unique metabolic structures (Fig. 2f), its predictions are more often redundant. We also found that MicrobeRX predicted a larger number of enzymes to metabolize drugs compared to BioTransformer (Fig. 2g, Supplementary Table 5).

Encouraged by these results, we decided to explore the potential of MicrobeRX to predict a list of well-curated drug metabolites from the literature. For their SIMMER tool, Bustion et al. [25] created a list of positive controls that are experimental gut metabolites that included data from experimental studies [25]. This list contains 18 entries indicating metabolic conversion of orally administered drugs, for which we found 13 exact matches in our predictions, including 9 with scores higher than 1.2 (Supplementary Table 8). For most of these correct predictions, the reaction in MicrobeRX is closely related to the one reported in the literature. One example here is the conversion of L-dopa to dopamine by *Enterococcus faecalis* and further to m-tyramine by *Eggerthella lenta* A2, a two-step metabolic transformation [55]. With MicrobeRX we indeed found the corresponding metabolite for the first conversion of l-dopa to dopamine, with a high confidence score (1.927). We also found that the enzyme responsible can be human and microbial l-phenylalanine carboxy-lyase (PHYCBOXL, EC: 4.1.1.28, 4.1.1.53), while tyrosine decarboxylase (TDC, EC: 4.1.1.25) was reported to be the responsible enzyme [55]. These two enzymes, however, belong to the aromatic-l-amino-acid decarboxylase protein family, which has closely related enzymatic functions and structures (InterPro: IPR010977). While we did not find PHYCBOXL in *E. faecalis*, we did find it in a related organism from the same genus, *Enterococcus cecorum* DSM 20682.

In summary, our tool significantly improved the number of enzyme annotations included in the predictions for both human and gut microbes (Fig. 2g). While BioTransformer frequently uses non-specific annotation, for example when referring to the CYP450 family, MicrobeRX can provide detailed enzyme names and pathways. Furthermore, BioTransformer lacks information about the source organisms for gut microbial transformations, rendering it ineffective for identifying direct links between metabolites, enzymes, and bacteria. One important consideration when predicting metabolites is the use of scoring metrics to evaluate and reduce false positives. In this regard, while MicrobeRX processed fewer drugs, its confidence score enables the prioritization of metabolites for further investigation.

These findings demonstrate that the metabolites predicted by MicrobeRX show properties similar to those found in biological samples and that its prediction outperforms BioTransformer in a number of results, molecular diversity, reduction of redundant predictions, and enzyme annotation.

#### Drug metabolism by human and gut microbes

**Fig. 3:**
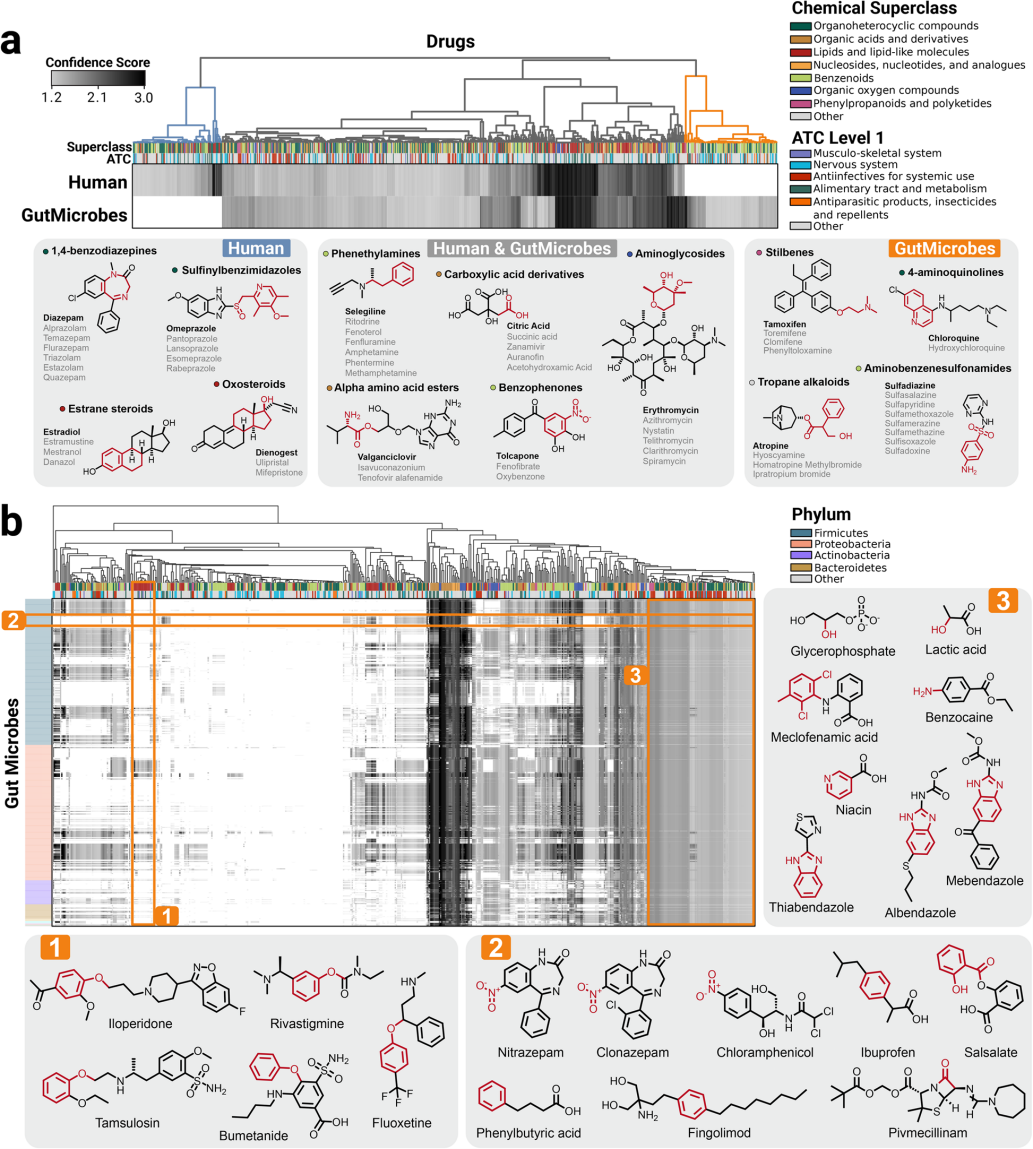
Metabolizing patterns of gut microbes from the best predictions of MicrobeRX.** a** General overview of drug distribution with the top best predictions for humans (blue), gut microbiota (orange), and both (gray). The top best predictions correspond to the high-confidence prediction (confidence score cutoff of ≥ 1.2) with the highest confidence score per drug–microbe pair. **b** The metabolic landscape of 6,286 strains included in MicrobeRX and what they can metabolize. The highest confidence scores for the prediction of each drug are plotted, showing both chemo-specific (group 1) and taxa-specific (group 2, strains of *Bacillus cereus* in the plot) metabolism patterns, as well as drugs that can be extensively metabolized (group 3). The heatmap is ordered based on clustering the drugs by the top best confidence score. The *y*-axis is organisms arranged alphabetically by phylum. The horizontal colored bars at the top of both plots indicate the drug superclass chemical classification and the anatomical therapeutic chemical (ATC) classification level 1. The molecules in the gray boxes exemplify the chemo-, taxa-, and extended-metabolism patterns. An example of the substructure modified during metabolism is colored in red

To delve deeper into the MicrobeRX predictions and provide a general overview of drug biotransformation, we narrowed our initial selection to the top best predictions based on GEM-CSRRs, yielding 106 predictions for gut microbes, 102 for humans, and 526 for both (Fig. 3a**, **Supplementary Table 9).

For the drugs that can be metabolized by both human and gut microbes, we observed that they share structural similarities with endogenous nutritional compounds such as lipids, particularly vitamin A, vitamin D, and steroid-related compounds. We also observed several carbohydrate and amino acid derivative drugs, including selegiline (treatment for major depressive disorder and Parkinson’s disease), erythromycin (macrolide antibiotic), and valganciclovir (antiviral against cytomegalovirus). Interestingly, drugs with pyrimidine rings are often predicted to be metabolized by both human and microbial enzymes. Examples include nicotine (stimulatory alkaloid, Fig. S9a) and 5-fluorouracil (5-FU) of tegafur [56] (Fig. S9b), previously reported to be metabolized by humans and gut bacteria by the same metabolic pathways. Tegafur is a 5-FU prodrug with antineoplastic properties used to treat advanced gastric and colorectal cancers [57, 58].

Top predictions for the gut microbiome were found particularly for organoheterocyclic, organic oxygen, and benzenoid drugs with various therapeutic functions. We observed that the drugs enriched in this category were treatments for nervous system disorders and anti-infectives (sulfadiazine, a sulfonamide antibiotic, Fig. S10a) and antiparasitics (chloroquine, an antimalaria drug), probably supporting the presence of resistance mechanisms in gut bacteria against these therapeutic agents [59–61]. Intriguingly, MicrobeRX predicted two metformin metabolites derived from *Citrobacter freundii* P-ribosyl-[dinitrogen reductase] that could use this drug as a substrate to produce the corresponding ADP-D-ribose-metformin product (Fig. S10b). While several studies have highlighted the importance of metformin in shaping the gut microbial composition of type 2 diabetes patients [62–65], the underlying molecular-level mechanism is still unknown [66].

For human metabolism, the predictions with higher scores corresponded to estrane steroids and oxosteroids such as estradiol (hormone replacement therapy, Fig. S11a) and dienogest (treatment of endometriosis and a contraceptive). This is in line with extensive evidence supporting the oxidative metabolism of estrogens by human cytochrome P4503A4/5 in liver cells [67, 68]. In addition, proton pump inhibitor drugs such as omeprazole and pantoprazole were predicted to be metabolized by CYP2C19, as previously reported [69]. Importantly, our analysis shows that MicrobeRX can also aid in the study of drugs for which there is limited information about their metabolism. For example, our results suggest that midodrine (alpha-adrenergic agonist used to treat orthostatic hypotension) and verapamil (anti-arrhythmia agent) might be metabolized by 3-methoxytyramine: oxygen oxidoreductase (Fig. S11b), which is thought to be present mostly in plasma [70] and liver [71].

#### Microbial drug metabolism exerts chemo- or taxa-specific patterns

We further zoomed in on microbial drug metabolism and found three distinct patterns: (1) *chemo-specific metabolism*, where bacteria can only metabolize specific drug classes or substructures with a specific chemical structure; (2) *taxa-specific metabolism*, where the conversion of drug groups is specific to phyla or genera; and (3) *extended-metabolism*, which is the conversion of drugs by almost any bacteria. We observed these three distinct patterns across various chemical classes and therapeutic applications in the drug dataset we explored (Fig. 3b).

One example of chemo-specific metabolism is the conversion of the anisole group present in drugs with diverse therapeutic functions, including iloperidone (antipsychotic for acute schizophrenia), tamsulosin (treatment for benign prostatic hyperplasia), and rivastigmine (cholinesterase inhibitor for Alzheimer’s and Parkinson’s disease). For these drugs, we found that the digallate acylhydrolase enzyme from the aminobenzoate degradation pathway may be involved in the hydrolysis of an ester of the anisole group present in the drugs. This enzyme was found in 273 strains from 20 different species of Proteobacteria and Firmicutes, with *Bacillus cereus* (117 strains) and *Klebsiella pneumoniae* (75 strains) being the most representative (Fig. S12a).

For taxa-specific metabolism, some interesting examples are the nitroaromatic compounds, such as nitrazepam and clonazepam (used to treat panic disorders, severe anxiety, and seizures) and chloramphenicol (a broad-spectrum antibiotic), which were primarily found for 1260 strains of Proteobacteria via periplasmatic nitroreduction. *Escherichia coli* (808 strains), *Salmonella enterica* (310 strains), and *K. pneumoniae* (78 strains) were predicted to be the most common bacteria involved in metabolizing these drugs (Fig. S12b). Microbial nitroreductases are gaining interest for their applications in the biotechnological, biomedical, and environmental fields for the synthesis of pharmaceutical precursors [72] and biodegradation of nitro-pollutants [73]; however, the physiological role of these enzymes, particularly in drug metabolism, is also a hot topic of study [73–78].

The predictions of 34 benzene-containing drugs, including the blockbuster drug ibuprofen (NSAID and non-selective COX inhibitor used to treat mild-moderate pain, fever, and inflammation), are another interesting example of taxa-specific metabolism. We found that the phenylpropanoate dioxygenase (EC 1.14.12.19) present in pathogens such as *E. coli* (790 strains), *Shigella flexneri* (14 strains), *Yersinia enterocolitica* (12 strains), and *Citrobacter freundii* (7 strains) may perform the dihydroxylation of the benzene ring of these drugs (Fig. S12c). Importantly, this enzyme has been extensively studied in *E. coli* for its function in using several aromatic acids as sole sources of carbon for growth [79], but little is known about how this enzyme may participate in the metabolism of ibuprofen or other benzenoid drugs [80].

Lastly, similar to the shared metabolism between human and gut microbes, the extended-metabolism of orally administered drugs was found mainly for endogenous or endogenous-like compounds. This might reflect the fact that certain drugs share structural patterns with substrates of enzymes present in almost all bacteria. For example, lactic acid (metabolic alkalinizing), glycerophosphate (sodium, used to treat hypophosphatemia), benzocaine (local anesthetic, Fig. S13a), and niacin (vitamin B) are metabolized by enzymes belonging to the pyruvate, glycerophospholipid, tryptophan, and NAD metabolism pathways, respectively. Interestingly, our results also indicate that benzimidazole antiparasitics such as albendazole, mebendazole, and thiabendazole could also be metabolized by the NAD metabolism pathway via nicotinate-nucleotide dimethylbenzimidazole phosphoribosyltransferase (EC 2.4.2.21), based on the similarity of these compounds to the enzyme–substrate 5,6-dimethylbenzimidazole, resulting in the potential formation of the α-ribazole-phosphate form of the drugs (Fig. S13b). This enzyme is also present in 6179 of the 6286 microorganisms included in MicrobeRX.

Furthermore, because our tool not only provides relationships but also allows for deep exploration of metabolic conversion mechanisms, we present some case studies that can help the scientific community better understand the enzymes involved in the conversion of relevant therapeutic drug classes.

#### Case studies

**Fig. 4:**
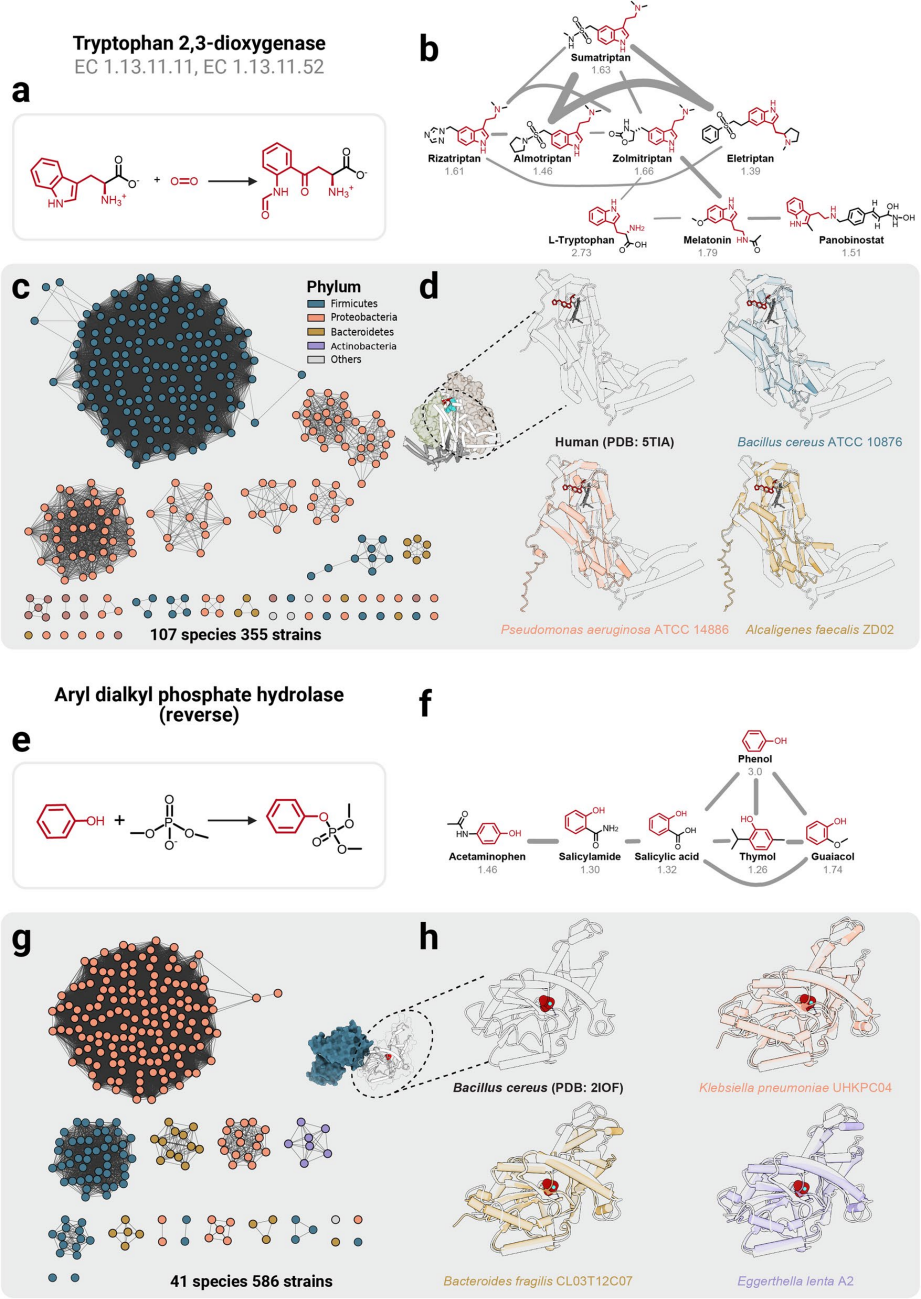
Shared and microbial-specific drug metabolism by human and gut microbes.** a** Shared metabolism of triptan drugs by human and microbial Tryptophan 2,3-dioxygenase (TRPO2, EC 1.13.11.11). **b** Molecular similarity network (MSN) of triptan drugs such as sumatriptan, zolmitriptan, and melatonin. **c** Protein sequence similarity network (SSN) of bacterial TRPO2. **d** Three different structural alignments of diverse microbial TRPO2 monomeric models and human TRPO2 (PDB: 5TIA), including the predicted binding mode of rizatriptan (red sticks) by molecular docking in the context of the HEME cofactor (gray sticks). **e** Microbial-specific metabolism by microbial aryl-dialkyl phosphate hydrolase (ADAPH, EC 3.1.8.1). **f** MSN of aryl alcohol–containing drugs, such as acetaminophen, salicylic acid, and guaiacol. **g** Protein SSN of ADAPH. **h** Three different structural alignments of diverse microbial ADAPH monomeric models against the ADAPH from *Bacillus cereus* (PDB: 2IOF), including the crystallographic mode of a phosphate ion (red spheres) in the context of the Mg^2+^ cofactor (cyan sphere). In the MSNs, edge size corresponds to the similarity between molecules at a 50% cutoff for constructing the trees. The SSN for both proteins was constructed using a 70% sequence similarity. Nodes and protein structures are colored according to phylum. The maximum common substructure of the drugs is colored in red

We further explored two different scenarios from the predictions from MicrobeRX: shared metabolism by human and gut microbes (Fig. 4a) and drug metabolism by microbes only (Fig. 4b). Here, we chose to focus on high-confidence predictions, using available structural information about the enzyme responsible to assess whether our predictions were sound from a structural standpoint. We also focused on common drugs—the triptan family for human and gut microbial metabolism and acetaminophen for microbial biotransformation.

In the first case, we found that the tryptophan 2,3-dioxygenase (TRPO2, EC: 1.13.11.11) present in humans and 107 species of gut microbes can incorporate an oxygen molecule into the indole side-chain of tryptophan-like molecules, using HEME as a cofactor, causing the pyrrole ring to open. We predict that this conversion can be carried out for triptan drugs used to treat migraine, such as zolmitriptan, sumatriptan, almotriptan, and rizatriptan, as well as for panobinostat (multiple myeloma antineoplastic agent) and melatonin (sleep–wake cycle regulator). Importantly, steady-state kinetic studies have shown that TRPO2 requires oxygen to catalyze the breakdown of tryptophan [81]. Nonetheless, TRPO2 may work intermittently in microenvironments with limited oxygen where small amounts of oxygen may diffuse in from host tissues [82]. In addition, some gut bacteria are facultative anaerobes, which may allow them to express and use TRPO2 when oxygen is available [83].

The protein sequence similarity network (SSN) of bacterial TRPO2 revealed that different bacterial phyla shared at least 70% sequence similarity between different strains (Fig. 4c). In contrast, the identity of human (RefSeq protein: NP_005642.1) and bacterial (RefSeq protein: WP_033682841.1) TRPO2 was found to be approximately 40%. TRPO2, despite its low sequence conservation, is a homo-tetrameric enzyme with 35–45 kD monomers and a fold and active site that is highly conserved from bacteria to humans [84]. Molecular and crystallographic studies support a two-step ferryl-based dioxygenation catalytic mechanism [84]. This enzyme is widely distributed in bacteria because it is required in the kynurenine pathway for tryptophan utilization, with 95% of tryptophan in the cell metabolized in this manner. Despite evidence linking the kynurenine pathway and migraines, little is known about the potential role of TRPO2 in triptan drug metabolism [85–87]. Our metabolic predictions and molecular docking results support the hypothesis that TRPO2 can bind and open the pyrrole ring of triptan drugs (Fig. 4d), and these initial results can serve as a starting point to study the effect of gut microbial metabolism in the host’s response to migraine treatments.

For the second case, we examined the metabolic capacities of microbial aryl-dialkyl phosphate hydrolase (ADAPH, Fig. 4e). This enzyme is commonly produced by bacteria isolated from soil samples, allowing them to degrade organophosphate compounds such as pesticides [88]. Interestingly, ADAPH has been reported to catalyze the reverse reaction (EC: 3.1.8.1), where a dialkyl phosphate and aryl alcohol are bound to produce an aryl-dialkyl-phosphate product. We found ADAPH in 41 different microbial species. We predicted that this enzyme could participate in the metabolism of six aryl-alcohol-containing compounds: phenol (antiseptic and disinfectant), salicylic acid (treatment for acne, psoriasis, and keratosis), salicylamide (analgesic and antipyretic), guaiacol (disinfectant and expectorant), thymol (antibacterial and antifungal), and acetaminophen (analgesic, Fig. 4f).

We noticed that the protein SSN of ADAPH shared at least 70% sequence similarity between different strains (Fig. 4g). We therefore generated the protein structures for some of these bacteria and discovered that the model from *Bacillus cereus* ATCC 4342 (WP_000861863.1) closely matched the crystallographic structure of phosphonoacetaldehyde hydrolase of the same organism (Protein database (PDB): 2IOF, similarity 95.6%), allowing us to make some structural inferences. Experimental evidence indicates that this protein forms a dimer and uses Mg^2+^ as a cofactor to perform an acid–base catalysis via Schiff-base formation^89^. In bacteria, this enzyme is found to convert the insecticides paraoxon and parathion into 4-nitrophenol in the aminobenzoate degradation pathway^90^. No information is available about bacterial ADAPH in the metabolism of aryl alcohol drugs. In this case, our results and binding predictions can serve as a starting point to study the influence of this enzyme in the metabolism of blockbuster drugs such as acetaminophen (Fig. 4h).

Altogether, these findings highlight how MicrobeRX can provide insight into the specific organisms and enzymes that may be responsible for the metabolism of orally administered drugs, as well as predict their metabolic products. The diversity of the organisms and drugs included in this screening may serve as a starting point for validating these metabolic conversions and for understanding the full potential of microbial organisms in health and various disease states.

## Discussion

The primary objective of this study was to investigate the molecular mechanisms of drug metabolism by gut microbiota. One of the most significant obstacles to advancing our understanding of gut microbiome metabolism is the vast chemical space of potential metabolites and the complexity of the enzymatic reactions encoded by gut microbes [91]. To address this problem, we developed MicrobeRX, a knowledge-based tool for studying xenobiotic metabolism by humans and gut microbes, and applied it to predict human-microbe drug metabolism of 1083 orally administered drugs from DrugBank.

Compared to existing protocols, MicrobeRX offers several advantages for predicting metabolites in terms of chemical diversity, the accuracy of the properties and structural fingerprints of reported metabolites, and the number of annotated references. Unlike other tools that focus on identifying reacting atoms or functional groups, MicrobeRX considers the chemical characteristics and connectivity of molecules. Additionally, our tool’s confidence scoring feature enables the selection of probable metabolites and provides evidence for the enzymes and organisms involved in biotransformation. To the best of our knowledge, no other approach integrates organisms, enzymes, and metabolites at the scale of MicrobeRX, making it a valuable tool for generating hypotheses aimed at discovering the metabolic conversion of large chemical classes.

However, MicrobeRX suffers from difficulties that are inherent to predicting metabolites, even when those metabolites have been discovered by experimental approaches. This is because, even for a newly discovered biotransformation, the chemistry and the enzymes responsible might still be under study or remain unknown. For example, while two enzymes may perform hydroxylation, their recognition of molecular patterns on the substrate, the substrate binding sites, the cofactors required, and the catalytic residues may differ significantly, resulting in distinct reaction outcomes. Not knowing these detailed chemical reactions reduces the predictive power of tools such as MicrobeRX. Besides, while GEMs provide a substantial source of biotransformations, our predictions depend on the availability of well-reported reactions in GEMs. Reactions not available or not fully described will be missing from our reaction rule database. Despite this limitation, MicrobeRX offers an extensible framework that can expand the chemical knowledge used to predict novel metabolites, based on the best available reaction data. This is the main value of our tool as a resource for generating hypotheses and discovering novel metabolites, even with its current limitations. Our *RuleGenerator* module also allows users to expand reaction rules of interest to them.

Furthermore, while several experimental attempts have been made to identify drug metabolites produced by gut bacteria, e.g., Zimmermann et al. 2019 [10], technical challenges hampered our efforts to use these results to validate MicrobeRX. Zimmerman et al. examined 76 bacterial strains (42 found in AGORA2), but none of the 30 gene products they identified could be remapped due to out-of-date protein IDs. For example, the diltiazem-converting protein BT_4096 in *Bacteroides thetaiotaomicron* VPI-5482 (originally reported as a hypothetical protein, current ID: WP_008760980.1) is not present in its GEM in AGORA2. This is because the relevant reactions of drug biotransformations are not yet included in AGORA2’s metabolic reaction database [32], indicating a need for future updates to incorporate these transformations into MicrobeRX. On the other hand, we were able to find the metabolites from other positive controls such as the conversion of L-dopa to dopamine [55], the deglycosylation of capecitabine and trifluridine [56, 92], and the nitro reduction of clonazepam and nitrazepam [93, 94] (Supplementary Table S8).

Similarly, identifying specific biotransformations in different organisms requires high-quality annotated genomes to generate GEMs, limiting the inclusion of some organisms. Furthermore, while our approach predicts metabolites from single bioconversion steps, predicting metabolites produced by metabolic pathways or cascades that involve different organisms remains challenging. Our CSRRs also cannot represent transport (transferases) or stereo conversion (isomerases) because these reactions do not alter the atom connectivity of the substrate. Future versions of MicrobeRX will focus on pathway connectivity to better predict metabolites and on models that can predict metabolite transport and stereo conversion. In addition, we would like to emphasize that MicrobeRX can only theoretically predict metabolic products if the enzyme is present in the genome. It also cannot quantitatively predict the transformation rate, which requires detailed information such as taxa abundance, enzyme abundance, activity, kinetics, and substrate concentration. Moreover, although MicrobeRX outperformed BioTransformer in the benchmarking analyses using DrugBank metabolites, the low overlap of GEM-based predictions between the two tools underscores the challenges in metabolite prediction and highlights the need for experimental validation.

Despite its limitations, MicrobeRX demonstrates considerable potential in predicting metabolites with properties similar to those found in biological samples, highlighting its utility in personalized nutrition, drug development, and pharmacotherapy.

Our analysis of human and microbial-specific drug metabolism revealed that drugs metabolized by both human and gut microbes often share structural similarities with endogenous compounds such as vitamins and amino acids. Examples include triptan drugs like zolmitriptan, which is predicted to be metabolized by both human and microbial TRPO2, and pyrimidine drugs like nicotine 5-FU, which is predicted to be metabolized by both human and microbial enzymes. This shared metabolism highlights the interplay between host and microbial biotransformations.

We also observed distinct patterns in microbial drug metabolism: chemo-specific metabolism, where bacteria metabolize specific drug classes or substructures; taxa-specific metabolism, where conversion is specific to a particular phylum or genus; and extended-metabolism, where almost any bacteria can convert the drugs. For instance, anisole group drugs are metabolized by digallate acylhydrolase found in Proteobacteria and Firmicutes, whereas nitroaromatic compounds are metabolized by periplasmatic nitroreductase in Proteobacteria. These findings emphasize the diverse metabolic capabilities of gut microbes.

The impact of microbial drug metabolism on drug efficacy and resistance mechanisms was another interesting finding in our study. In one example, metformin metabolism by *Citrobacter freundii* produces ADP-D-ribose-metformin, potentially influencing the gut microbial composition in type 2 diabetes patients. Such insights can serve as a starting point to dig deeper into projects to develop personalized medicine by enabling tailored drug therapies based on microbiome profiles and dosage adjustments to ensure the highest efficacy with the lowest adverse effects.

Overall, MicrobeRX is a versatile tool that can reveal how the gut microbiome affects nutrition and health by predicting the microbial metabolism of dietary components and drugs. Its application can lead to novel therapeutic strategies for various health conditions related to microbial imbalances, making it a relevant resource for exploring the complex metabolic landscape of the gut microbiome.

Because of its powerful prediction capabilities, MicrobeRX can extend beyond drug metabolism, offering significant potential for various research and industrial fields. First, the tool’s ability to identify novel enzymes catalyzing unique biochemical reactions makes it invaluable for enzyme discovery and synthetic biology. For instance, researchers can harness MicrobeRX to pinpoint enzymes with desirable properties for industrial applications, or use it to design synthetic pathways for producing high-value chemicals. This capability can accelerate the development of synthetic organisms and engineered enzymes tailored for specific biotechnological purposes. Second, comprehensive metabolic predictions can help in generating hypotheses for exploring new chemical classes in various applications. In bioremediation, MicrobeRX can identify microbial strains capable of degrading environmental pollutants, aiding in the development of effective clean-up strategies. In agriculture, it can inform sustainable practices by predicting microbial pathways that enhance nutrient availability or degrade agrochemicals. The applications of our tool also extend to the food industry and nutritional science, where it can be used to optimize fermentation processes or develop personalized nutrition strategies. Thus, MicrobeRX stands as a versatile resource for advancing research and innovation across multiple scientific and industrial fields.

## Materials and methods

### Inclusion of host and intestinal microbe metabolism in MicrobeRX

Recon3D was used as the source for human biotransformation (https://www.vmh.life/files/reconstructions/Recon/3D.01/Recon3D_301.zip), and 6286 strains with publicly available genomes and metabolic reconstruction in AGORA2 [32] were used as sources of microbial metabolism. All metabolic reconstructions were downloaded (https://www.vmh.life/files/reconstructions/AGORA2 version 2.01) and processed using cobrapy 0.26.2 [95]. Metabolic reactions, directions, compartments, subsystems (pathways), gene names, metabolites, and annotations were extracted for each reconstruction. For all reactions and the metabolites from the reconstructions, annotations were cross-referenced using MetaNetX v4.4 [37].

### Gene remapping

The majority of gene names from AGORA2 reconstructions contained gene IDs not found in the NCBI Reference Sequence Database (RefSeq, https://www.ncbi.nlm.nih.gov/refseq/-) or the Bacterial and Viral Bioinformatics Resource Center (https://www.bv-brc.org/). The reference genomes of the 6286 strains available in RefSeq were uploaded to Kbase (https://www.kbase.us/). The genomes were then reannotated using the *Annotate Multiple Microbial Genomes with RASTtk*—v1.073 tool, which preserved the gene IDs, before being processed with the *Build Multiple Metabolic Models* module to generate draft GEMs. Finally, the AGORA2 reactions were compared to our draft reconstructions. To facilitate further analysis with MicrobeRX, all gene names were remapped as RefSeq protein IDs. The Kbase workflow with detailed steps is available at: https://narrative.kbase.us/narrative/164491. In this way, we successfully mapped 4,606,131 RefSeq protein IDs for 3224 microbial reactions.

### Generation of biotransformation-based reaction rules

The CSRRs for each single reactant reaction (SRR) were generated using the RDKit cheminformatic toolbox (http://www.rdkit.org/), as previously reported [43]. This functionality is embedded in the MicrobeRX *RuleGenerator* module, and the procedure is described below in brief.

1. As we were focused on reactions that involved atomic changes, we excluded transport, exchange, passive diffusion, biomass production, and stereochemical conversion reactions. We further filtered out unbalanced reactions and reactions with missing reactants or products.
2. All compounds involved in the reactions underwent molecular sanitization, which included replacing dummy atoms (R-groups) with carbons, setting explicit aromaticity to Kekule form, removing stereochemistry, and adjusting the valency of all the atoms to neutrality.
3. Sanitized reactions with < 1200 atoms were then mapped using RXNMapper [96], a neural-network-based program for atom-mapping of chemical reactions, or with the Reaction Decoder tool (RDT), an open-source software for mining reaction features and centers and calculating the similarity between reactions [97].
4. To identify the atomic changes between each substrate and all biotransformation products, we decomposed each reaction into SRRs. An SRR is a computational method that iteratively matches atom changes in each substrate to all products in a chemical reaction. As a result, each biotransformation generates a number of SRRs that is equal to the number of substrates in the reaction. This method is comparable to RetroRules, a server containing reaction rules for the discovery and engineering of metabolic pathways [43].
5. Each SRR was then converted into a series of CSRRs by determining the atomic differences between the substrate and the products. Detailed atom connectivity, ring membership, aromaticity, and charge were included in the atomic descriptions of reactants and products in order to provide the CSRR with a high level of chemical information for prediction.
6. Finally, CSRRs were generated by iterating and trimming over the reacting and neighboring atoms until the substrate contained either up to 40 atoms or all the substrate atoms. Each CSRR was then saved in SMILES ARbitrary Target Specification (SMARTS) notation, and the main substrate and product of the SRR were saved as Simplified Molecular Input Line Entry System (SMILES) notation, for later use in the metabolite predictor to calculate the confidence score.

### MicrobeRX development

Using the CSRRs generated from the reactions in the GEMs, we developed a Python library to perform predictions that includes the following modules (Fig. S5):

1. **Rule generator**. This module contains all the functions required to automatically generate CSRRs. It accepts a reaction scheme from GEMs or databases as input, as well as a list of substrates and products involved in the reaction. *RuleGenerator* can then decompose reactions, map them, create SRRs, and generate CSRRs (Fig. S2). The CSRRs from the Recon3D and AGORA2 GEMs are saved to a database for use in the *MetabolitePredictor* module (https://microberx.readthedocs.io/en/latest/autoapi/microberx/DataFiles).
2. **Metabolite predictor**. This module makes use of the CSRR generated by the *RuleGenerator* module and the RDKit toolbox. Users can choose whether to apply all reaction rules or to focus on a specific biosystem (human or gut microbes). Furthermore, given the possibility of false positives, it allows users to set a confidence score cutoff for the predictions (default 0.6). The predictor then matches the query to the substructure from the real substrate in the CSRR for each rule. If a match is found, the predictor executes the chemical conversion specified in the CSRR. The main product is then considered the predicted metabolite. However, our approach also provides secondary products of reactions, which frequently correspond to cofactors, carbon dioxide, water, and molecular oxygen. The confidence score of the predicted metabolite is then calculated (Fig. S3). Finally, following the predictions, *MetabolitePredictor* provides information from the GEMs about reaction ID, enzyme, pathway, compartment, biosystem, atom-mapping for metabolic accessibility, and several database annotations of the reaction (https://microberx.readthedocs.io/en/latest/autoapi/microberx/MetabolitePredictor).
3. **Metabolite analyzer**. MicrobeRX includes several automated and optional analyses for investigating the chemical and structural properties of predicted metabolites. Once the predictions have been made, the analyzer can use PubChemPy 1.0.4 (https://github.com/mcs07/PubChemPy) to search PubChem for annotation on the predicted structures. It can use RDKit to compute molecular descriptors such as Pan-assay interference compounds (PAINS) [98] and identify substructures with undesirable pharmacokinetics or toxicity (Brenk) [99], as well pyOpenMS 2.4 [46] to compute isotopic mass decomposition and ClassyFire [47] to generate an automated chemical classification.
4. **Metabolite visualizer**. After the analysis is completed, the *MetaboliteVisualizer* module can display interactive plots using Plotly to show relevant chemical characteristics of the predicted metabolites. This module can display the metabolic accessibility of the query, which computes and graphically displays the frequency of each atom to match with the substructures in the CSRRs during prediction. *MetaboliteVisualizer* also allows visualization of the predicted metabolite structures in an ordered manner, including relevant annotations when available, and a 3D scatter displaying the different components of the confidence score. Next, it can show a spider plot of physicochemical properties including the Lipinski’s rule of five thresholds, a plot with the predicted gastrointestinal absorption and brain penetration using the BOILED-Egg method [48], and a bar plot of the isotopic mass decompositions. Finally, *MetaboliteVisualizer* can show a customizable Sankey plot of the relationships between predicted metabolites, linked with enzyme names, origin, and other information from the results table (Fig. S6).
5. **Omics integrator**. Finally, MicrobeRX includes functionalities for connecting predictions to gene information from GEMs. For each metabolite, this module can show an interactive sunburst plot of the microbial species that produce it. Using the RefSeq protein ID included in our tool, MicrobeRX can automatically retrieve a list of sequences by implementing Biopython [100]. These sequences can then be compared to generate an interactive heatmap of the pairwise similarity scores for a given set of sequences, as well as to perform an MSA and phylogenetic tree construction with ClustalW2 [101]. It can also generate an MSA chart and display the degree of conservation between sequences. Finally, for selected sequences, it can produce a bar plot of the protein families and domains by performing an InterProScan [51] search (Fig. S7).

The MicrobeRX code, including installation details and tutorials, is available at (https://microberx.readthedocs.io/en/latest/). We have also created a standalone server where users can perform MicrobeRX predictions and analysis without installation (https://colab.research.google.com/drive/1bELtC9POifs8ExVqHDoVEu_yaBeWcF8Y?usp=sharing).

### Confidence score

Once a prediction is made using the CSRR, the confidence score is computed. This score is comprised of three parameters: (i) the molecular similarity between the query for the prediction and the substrate represented in the CSRR, (ii) the molecular similarity between the prediction produced and the original product represented in the CSRR, and (iii) the ratio of the number of atoms in the substructure that match between CSRR and query to the total atoms in the query (atom efficiency). As a result, higher confidence scores directly reflect the likelihood that a predicted metabolite exists and that the reaction involved is the true mechanism of its chemical conversion. Lower confidence scores may indicate a higher rate of false positive predictions. Finally, as the confidence score is the sum of the three parameters, it has a maximum value of three. The molecular similarity is calculated using RDKit and RDKFingerprints of 2048 bits generated from 1 to 7 bond paths. We suggest using a confidence score cutoff of 1.2, as this represents a minimum of 40% molecular similarity between the query and the real substrate.

#### Drug selection, metabolite prediction, and cheminformatic analysis

We selected orally administered approved drugs from the DrugBank database v.5.1.10 (Supplementary Table 4) [34]. The drug structures were sanitized prior to prediction by removing salts, neutralizing charges, and adjusting valences using DataMol 0.11.4 [102]. Metabolites for each drug were then predicted using the *MetabolitePredictor* module using the rules for the human and gut microbes (biosystem = ”all”), and predictions were saved if confidence_score ≥ 0.6 (cut_off = 0.6). The sanitized drug structures were also submitted to BioTransformer 3.0 to predict human and gut microbiome metabolites, using the default parameters *all human* or *hgut* biotransformation types for human or human gut microbial prediction, respectively. The predictions for each drug and biotransformation type were saved for further analyses.

For the MiMeDB [35] metabolites, we selected entries annotated as “detected and quantified,” “detected but not quantified”, and “expected but not quantified” from biological matrices (blood, urine, saliva, feces, sweat, and bile) and exogenous sources (food, plant, toxin/pollutant, cosmetic, microbial, drug, and drug metabolites). This yielded 24,039 unique compounds out of 27,641 metabolites (https://mimedb.org/, accessed in August 2023).

To identify the exact structures overlapping between the results from MicrobeRX, BioTransformer 3.0, MiMeDB, and DrugBank and perform a robust comparison, we computed Self-Referencing Embedded Strings (SELFIES) [103]. The physicochemical descriptors of the non-redundant molecules of all datasets were also calculated using the MicrobeRX *MetaboliteAnalyzer* workflow. Structural diversity was assessed by principal component analysis on Morgan fingerprints using 2048 bits and a radius of at least two bonds computed with RDKit.

The results of the MicrobeRX drug metabolite predictions, as well as a workflow analysis, are accessible at https://colab.research.google.com/drive/170rIHrZDpXxaL7HjAD-v1LfU5yjADtva?usp=sharing.

#### Molecular similarity network (MSN)

To assess the similarity relationships between a group of selected drugs and drug metabolites, the molecular similarity was computed using Morgan fingerprints, as described above. The similarity matrix was then used to create a network using the molecules as nodes and the similarity between them as edges. This network was visualized with Cytoscape 3.10.2 [104].

#### Sequence similarity networks (SSNs)

Protein SSNs were built like the MSN. Protein sequence similarity between protein sequences was calculated using the ClustalW 2.0 [96] program in the *OmicsIntegrator* module of MicrobeRX. The similarity matrix was then used to create a network using the IDs of sequences as nodes and their similarity as edges. The network was visualized with Cytoscape 3.10.2 [104].

#### Protein modeling and structure comparison

The human structures of Cytochrome P450 1B1 (reaction ID: P4501B1, RefSeq ID: 1545, PDB: 6IQ5), 3-methoxytyramine oxidoreductase (reaction ID: 3MOXTYROX, RefSeq ID: 4128, PDB: 2Z5Y), and tryptophan 2,3-dioxygenase (reaction ID: TRPO2, RefSeq ID: 6999, PDB: 5TIA) were retrieved from the PDB and used for further analysis. Similarly, the structure of the aryl-dialkyl phosphate hydrolase from *Bacillus cereus* (reaction ID: ADAPH, PDB: 2IOF) was obtained from the PDB.

Due to the lack of experimental structural availability for the bacterial proteins, we used ESMFold [100] to model the structures of tryptophan 2,3-dioxygenase for *B. cereus* ATCC 10876 (WP_033682841.1), *Pseudomonas aeruginosa* ATCC 14886 (WP_003120044.1), and *Alcaligenes faecalis* ZD02 (WP_045929451.1) and those of the aryl-dialkyl phosphate hydrolase from *Klebsiella pneumoniae* UHKPC04 (WP_002923203.1), *Bacteroides fragilis* CL03T12C07 (WP_005790570.1), and *Eggerthella lenta* A2 (WP_015761084.1). The 3D structures were then refined by running a 1 ns relaxation simulation using the amber99sb forcefield via OpenMM [105]. Structural comparisons, analysis, and visualizations were conducted using UCSF ChimeraX 1.7.1 [106].

#### Molecular docking

We used molecular docking to represent the binding modes of selected drugs to catalytic enzymes. The proteins from crystal structures or generated models were prepared using LePro (http://www.lephar.com/) to remove co-crystallized waters and solvent molecules and add charges and hydrogens. Ligands were prepared by adding explicit hydrogens and tautomeric states at pH 7.4 and generating 3D coordinates using Standardized 19.20.0 (http://www.chemaxon.com). Smina [107] was used to perform the docking within a 4 Å box from identified catalytic sites, including cofactors if present. For each ligand, at least 50 poses were requested and saved for analysis. For each protein, the lowest root mean square deviation to the original co-crystallized ligand was used to determine the best result.

## Supplementary Information

Supplementary Material 1. Figure S1. Collective abundance of MicrobeRX species in the human gut microbiome. 6,286 strains could be classified into 1,396 species, of which 330 species are common in the human gut microbiome. Collectively, they account for 77% of total microbial composition on average in 8,482 metagenomic samples from the Dutch Microbiome project. X-axis refers to subject. Y-axis refers to the total proportion. Blue area indicates the collective proportion of the 330 matched species. Gray area indicates the abundance of other species. Subjects are ordered by the collective proportion of the matched species. Figure S2. Example of the use of the *RuleGenerator *workflow to produce CSRR from genome-derived. GEMs. To generate CSRR, chemical transformation information is required from GEMs based on annotated genomes (MetanetX, BiGG models, etc.) or reaction databases (MetaCyc, KEGG). The *RuleGenator *module of MicrobeRX begins generating CSRR by enumerating and describing all the atoms in the reaction, e.g., whether they belong to a ring and how they are connected to other atoms (top scheme). The atoms involved in the chemical change are then identified (reacting atoms), and the CSRR is generated by trimming the atoms surrounding the reacting atoms to produce shorter versions of the chemical transformation (lower panel). Finally, the CSRR are saved as SMARTS reaction strings (lower text) for interoperability with other cheminformatics tools. Hydrogen atoms have been removed from the schemes to improve clarity. Figure S3. Development and example of the confidence score. The primary goal of the confidence score is to determine whether a query and its predictions match the actual substrate and product of an enzymatic reaction. As a result, after predicting a query compound, the molecular similarity between the substrate and query (pink box) and that between product and prediction (green box) is calculated (both are between 0 and 1). The final component of the confidence score (atom efficiency, also between 0 and 1) evaluates the reliability of the CSRR used in the prediction by computing the ratio of the query's atoms that match the substructures in the CSRR (yellow highlighting). The confidence score is calculated by adding the substrate-query, product-prediction, and atom efficiency values. The two examples show the components of the confidence score for two structurally related queries that produce different confidence scores due to greater difference in atom efficiency between the molecules. Figure S4. Implementation of metabolic accessibility in MicrobeRX. For each prediction, MicrobeRX assesses the number of atoms that match between the CSRR and the query (Fig. S2). As a result, metabolic accessibility is defined as the frequency with which each atom is recognized in the CSRRs, which is represented by a color scale. In the case of hydrocortisone (DB00741), the atom pair C-OH, which corresponds to atoms 5 and 6, appears most frequently in all of the CSRRs used to predict this drug's metabolites. As a result, the hydroxyl group is highlighted as being more metabolically accessible than the other hydrocortisone atoms. Figure S5. Schematic representation of MicrobeRX Python library. Overview of the MicrobeRX Python library illustrating the integration of reaction data from GEMs of human and gut microbiomes and the various modules within MicrobeRX, such as *RuleGenerator *and *MetabolitePredictor *(green boxes). Figure S6. Example of analysis and visualization outputs from the *MetaboliteAnalyzer *and *MetaboliteVisualizer*modules for hydrocortisone. (a) Molecular structure of the query for the prediction (usually SMILES). (b) Per atom metabolic accessibility diagram. (c) Five top predicted metabolites, sorted by confidence score and including PubChem annotation and structural classification. (d) 3D scatter plot for the different component’s confidence scores (x: substrate similarity, y: product similarity, z: atom efficiency). (e) Lipinski’s rule of five bioavailability radar plot based in molecular descriptors (MolWt: molecular weight, LogP: octanol-water partition coefficient, TPSA: Topological Polar Surface Area, RotB: number of rotatable bonds, HBD: number of hydrogen bond donors, HBA: number of hydrogen bond acceptors). (f) ADMETox (BOILED-Egg) plot including the limits for Blood Brain Barrer and Human Intestinal Absorption accessibilities. (g) Bar plot of isotopic mass decomposition for two predicted metabolites. (h) Sankey diagram depicting relationships between the reaction, origin, enzyme name, and metabolite produced. Figure S7. Example of outputs from the *OmicsIntegrator *module. (a) Sunburst plot of gut microbiome organisms per reaction/enzyme. (b) Cluster map of pairwise protein sequence comparisons for homology analysis. (c) Multiple sequence alignment with the consensus sequence (protein logo) highlighting conserved regions (blue bars). (d) Protein family search results from InterPro from a selected sequence of acetyl-CoA: cortisol oacetyltransferase from gut microbes showing the distribution of protein domains. (e) Crystal structure of acetyl-CoA: cortisol acetyltransferase from *Salmonella typhimurium *(PBD: 8I06) complexed with CoA (blue), shown as a representative of the protein responsible for the metabolism of hydrocortisone. Figure S8. DrugBank metabolite dataset for MicrobeRX benchmark and predictions. (a) DrugBank v.5.1.12 contains 11,928 drug structures, of which only 954 drugs have reported metabolites. (b) Number of metabolites per drug reported in the DrugBank metabolites dataset. (c) Top 25 reported enzymes for the 3,650 metabolic reactions from the DrugBank database. (d) Confidence score distribution of the 480,458 MicrobeRX-predicted metabolites from 19,560 CSRRs derived from the 3,650 DrugBank reactions. Predictions with a confidence score above the upper whisker threshold (Q3 + 1.5*IQR ≈ 1.206) are considered high-confidence. (e) Proportion of correctly predicted metabolites from the whole DrugBank metabolites dataset. (f) Number of drugs processed depending on the number of atoms in the GEM-based CSRRs. (g) Number of metabolites predicted by the GEM-based CSRRs. (h) Confidence score values depending on the number of atoms used in the GEM-based CSRR for the predictions. Black dashed line indicates the selection confidence threshold of 1.2. Figure S9. Predicted microbial metabolism of pyrimidine-containing drugs including nicotine and 5-fluorouracil (5-FU) by human and gut microbes. (a) Top panel displays the Molecular Similarity Network (MSN) of pyrimidine drugs (including nicotine) biotransformed by Nicotinamide Ribotide (NMN) Synthetase (NMNS, EC: 2.4.2.12). Edge size is the similarity between molecules at a 40% cutoff for constructing the tree. (b) Top panel displays the MSN of pyrimidine drugs (including tegafur) biotransformed by the periplasmic Deoxyuridine Phosphorylase (DURIPP, EC: 2.4.2.1). Edge size is the similarity between molecules at a 75% cutoff for constructing the tree. Lower panels represent the predicted metabolites from drugs selected in the MSNs (gray circles in a and b). The confidence score for each prediction is shown above the reaction arrow. Cofactors and secondary products are not shown for simplicity. The RefSeq identifier for human and the number of bacterial species containing the reaction are shown as a sunburst plot of enzyme phyla distribution. The maximum common substructure of the drugs is shown in red. Molecular substructures in blue are the predicted modifications from MicrobeRX. Figure S10. Example of microbial drug metabolism. (a) MSN of sulfonamides metabolized by the sulfasalazine microbial azo-reduction (SSZ_AR_NADi). Edge size is the similarity between molecules at a 70% cutoff for constructing the tree. (b) Metformin metabolism by *Citrobacter freundii*, producing ADP-D-ribose-metformin. The figure includes the chemical structures of the drugs and their metabolites, with confidence scores for each prediction shown above the reaction arrows. Cofactors and secondary products are not shown for simplicity. The number of bacterial species containing the relevant enzymes is shown as a sunburst plot of enzyme phyla distribution. Maximum common substructures of the drugs are shown in red. Molecular substructures in blue are the predicted modifications from MicrobeRX. Figure S11. Examples of predicted human metabolism. (a) MSN of estrane-derivatives, including estradiol, by human CYP450 1B1 (EC: 1.14.14.1). Edge size is the similarity between molecules at a 50% cutoff for constructing the tree. (b) Predicted biotransformation of midodrine and verapamil by human 3-Methoxytyramine: Oxygen Reductase (3MOXTYROX, EC: 1.4.3.4). Confidence scores for each prediction are shown above the reaction arrows. Cofactors and secondary products are not shown for simplicity. For each enzyme, the lower panel includes the best molecular docking predicted pose of tasimelteon (red sticks, -9.6 kcal/mol) in the catalytic site of CYP450 1B1 (PDB: 6IQ5) and midodrine (red sticks, -7.5 kcal/mol) in 3MOXTYROX (PDB: 2Z5Y). Maximum common substructure of the drugs is shown in red. Molecular substructures in blue are the predicted modifications from MicrobeRX. Figure S12. Examples of chemo- and taxa-specific microbial metabolism. (a) Metabolism of drugs containing anisole groups by digallate acylhydrolase (DGLTAH) in Proteobacteria and Firmicutes. Edge size is the similarity between molecules at a 50% cutoff for constructing the tree. (b) Metabolism of nitroaromatic drugs by periplasmatic nitroreductase (NRepp) in Proteobacteria, highlighting the prevalence of this enzyme in specific bacterial strains. (c) Biotransformation of benzene-containing drugs by phenylpropanoate dioxygenase (PPPNDO, EC: 1.14.12.19) in pathogens such as *Escherichia coli *and *Shigella flexneri*, with detailed structural changes during the metabolic process. Edge size is the similarity between molecules at a 55% cutoff for constructing the tree. Confidence scores for each prediction are shown above the reaction arrows. Cofactors and secondary products are not shown for simplicity. The number of bacterial species containing the relevant enzymes is shown as sunburst plots of enzyme phyla distribution. Maximum common substructures of the drugs are shown in red. Molecular substructures in blue are the predicted modifications from MicrobeRX. Figure S13. Examples of extended metabolism by gut microbiota. (a) Metabolism of endogenous-like compounds, including benzocaine by microbial Anthranilate phosphoribosyltransferase (ANPRT, EC: 2.4.2.18). Edge size is the similarity between molecules at 50% cutoff for constructing the tree. (b) Predicted biotransformation of benzimidazole antiparasitic by the Nicotinate-nucleotide dimethylbenzimidazole phosphoribosyltransferase (NNDMBRT) from the NAD metabolism pathway, showing the potential formation of α-ribazole-phosphate forms. Edge size is the similarity between molecules at 40% cutoff for constructing the tree. The figure includes chemical structures, with confidence scores for each prediction shown above the reaction arrows. Cofactors and secondary products are not shown for simplicity. The number of bacterial species containing the relevant enzymes is shown as sunburst plots of enzyme phyla distribution. Maximum common substructures of the drugs are shown in red. Molecular substructures in blue are the predicted modifications from MicrobeRX.

Supplementary Material 2.

## Data Availability

Data is provided within the manuscript or supplementary information files. A standalone server where users can perform MicrobeRX predictions and analysis without installation is accessible at https://colab.research.google.com/drive/1bELtC9POifs8ExVqHDoVEu_yaBeWcF8Y?usp=sharing. The results of the MicrobeRX drug metabolite predictions, as well as workflow analysis, are accessible at https://colab.research.google.com/drive/170rIHrZDpXxaL7HjAD-v1LfU5yjADtva?usp=sharing
