# Supplementary material for: MicrobeRX: a tool for enzymatic-reaction-based metabolite prediction in the gut microbiome
Source: Microbiome. 2025 Mar 19;13:78. doi: 10.1186/s40168-025-02070-5 (PMC11921629; doi:10.1186/s40168-025-02070-5)
Supplement: Supplementary file 2 — Supplementary Material 1. Figure S1. Collective abundance of MicrobeRX species in the human gut microbiome. 6,286 strains could be classified into 1,396 species, of which 330 species are common in the human gut microbiome. Collectively, they account for 77% of total microbial composition on average in 8,482 metagenomic samples from the Dutch Microbiome project. X-axis refers to subject. Y-axis refers to the total proportion. Blue area indicates the collective proportion of the 330 matched species. Gray area indicates the abundance of other species. Subjects are ordered by the collective proportion of the matched species. Figure S2. Example of the use of the RuleGenerator workflow to produce CSRR from genome-derived. GEMs. To generate CSRR, chemical transformation information is required from GEMs based on annotated genomes (MetanetX, BiGG models, etc.) or reaction databases (MetaCyc, KEGG). The RuleGenator module of MicrobeRX begins generating CSRR by enumerating and describing all the atoms in the reaction, e.g., whether they belong to a ring and how they are connected to other atoms (top scheme). The atoms involved in the chemical change are then identified (reacting atoms), and the CSRR is generated by trimming the atoms surrounding the reacting atoms to produce shorter versions of the chemical transformation (lower panel). Finally, the CSRR are saved as SMARTS reaction strings (lower text) for interoperability with other cheminformatics tools. Hydrogen atoms have been removed from the schemes to improve clarity. Figure S3. Development and example of the confidence score. The primary goal of the confidence score is to determine whether a query and its predictions match the actual substrate and product of an enzymatic reaction. As a result, after predicting a query compound, the molecular similarity between the substrate and query (pink box) and that between product and prediction (green box) is calculated (both are between 0 and 1). The final component of the con [file 40168_2025_2070_MOESM1_ESM.pdf]

Supplementary Figures

MicrobeRX: A tool for enzymatic-reaction-based metabolite prediction in the gut microbiome

Table of Contents

Figure S1. Collective abundance of MicrobeRX species in the human gut microbiome 2

Figure S2. Example of the use of RuleGenerator workflow to produce CSRR from genome-derived GEMs 3

Figure S3. Development and example of the confidence score. 4

Figure S4. Implementation of the metabolic accessibility in MicrobeRX 5

Figure S5. Schematic representation of MicrobeRX Python library. 6

Figure S6. Example of analysis and visualization outputs from the MetaboliteAnalyzer module for hydrocortisone 7

Figure S7. Example of outputs from the OmicsIntegrator module. 8

Figure S8. Description of the DrugBank metabolites dataset used for benchmark and predictions from MicrobeRX 9

Figure S9. Predicted microbial metabolism of pyrimidine-containing drugs including nicotine and 5-fluorouracil (5-FU) by human and gut microbes 10

Figure S10. Example of microbial drug metabolism. 11

Figure S11. Examples of predicted human metabolism. 12

Figure S12. Examples of chemo- and taxa-specific microbial metabolism. 13

Figure S13. Examples of extended metabolism by gut microbiota. 14

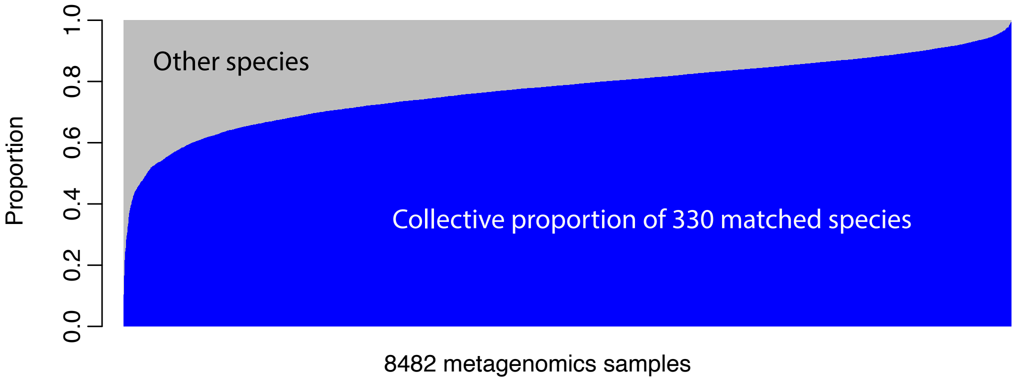

Figure S1. Collective abundance of MicrobeRX species in the human gut microbiome. 6,286 strains could be classified into 1,396 species, of which 330 species are common in the human gut microbiome. Collectively, they account for 77% of total microbial composition on average in 8,482 metagenomic samples from the Dutch Microbiome project. X-axis refers to subject. Y-axis refers to the total proportion. Blue area indicates the collective proportion of the 330 matched species. Gray area indicates the abundance of other species. Subjects are ordered by the collective proportion of the matched species.

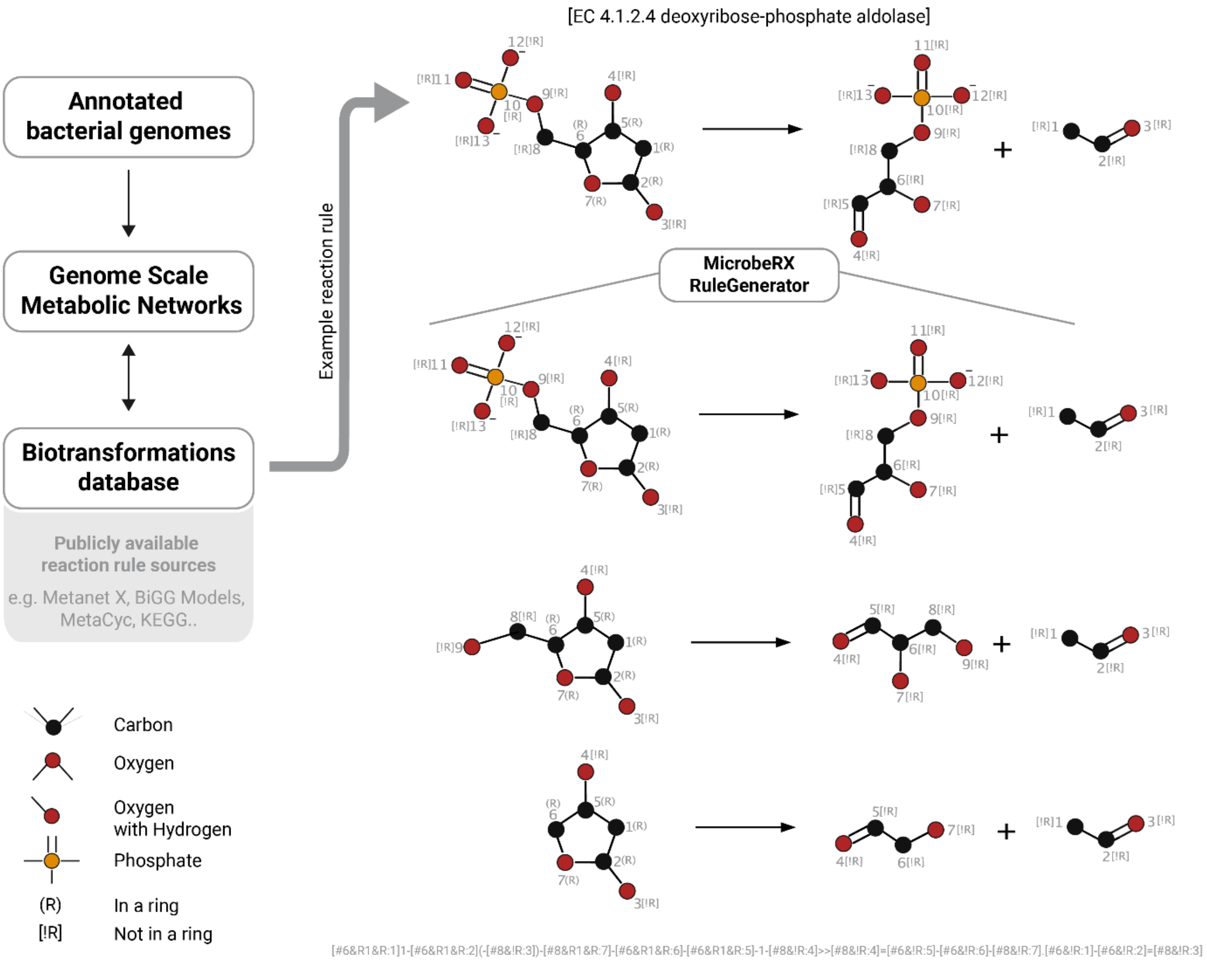

Figure S2. Example of the use of the *RuleGenerator* workflow to produce CSRR from genome-derived GEMs. To generate CSRR, chemical transformation information is required from GEMs based on annotated genomes (MetanetX, BiGG models, etc.) or reaction databases (MetaCyc, KEGG). The *RuleGenator* module of MicrobeRX begins generating CSRR by enumerating and describing all the atoms in the reaction, e.g., whether they belong to a ring and how they are connected to other atoms (top scheme). The atoms involved in the chemical change are then identified (reacting atoms), and the CSRR is generated by trimming the atoms surrounding the reacting atoms to produce shorter versions of the chemical transformation (lower panel). Finally, the CSRR are saved as SMARTS reaction strings (lower text) for interoperability with other cheminformatics tools. Hydrogen atoms have been removed from the schemes to improve clarity.

a

$$\text{Atom Efficiency} = \frac{\text{Query Matching Atoms}}{\text{Query Total Atoms}}$$

Confidence Score = **Substrate-Query similarity** + **Product-Prediction similarity** + **Atom Efficiency**
(maximun value ≤ 3)

b

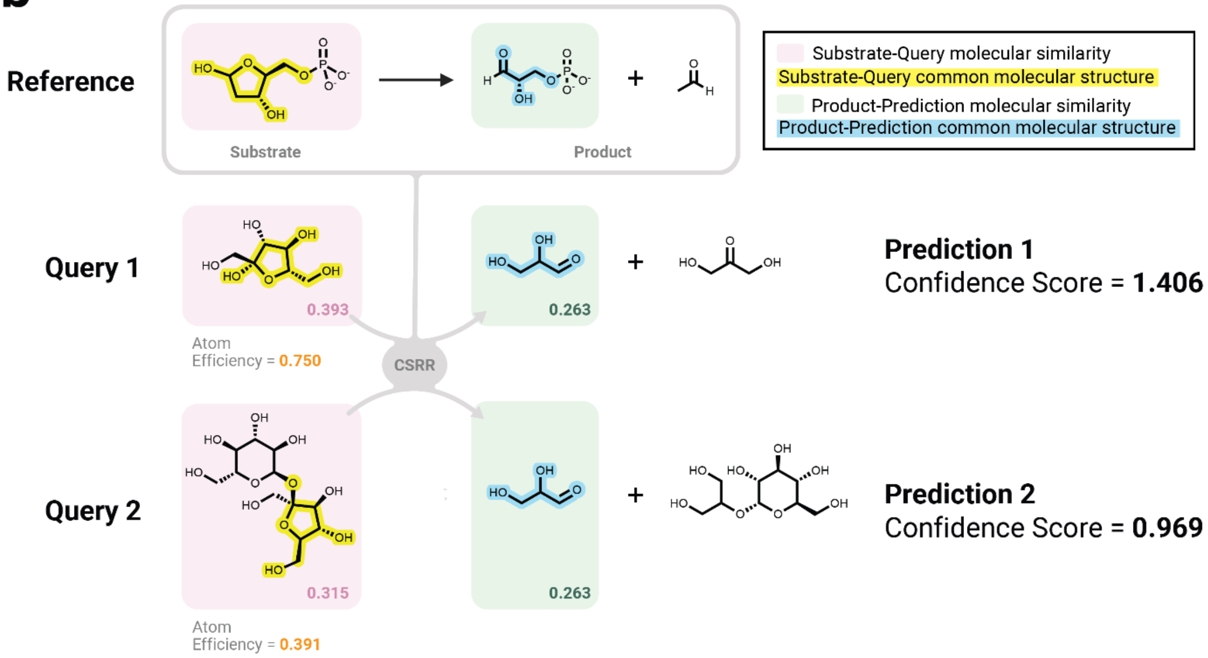

Figure S3. Development and example of the confidence score. The primary goal of the confidence score is to determine whether a query and its predictions match the actual substrate and product of an enzymatic reaction. As a result, after predicting a query compound, the molecular similarity between the substrate and query (pink box) and that between product and prediction (green box) is calculated (both are between 0 and 1). The final component of the confidence score (atom efficiency, also between 0 and 1) evaluates the reliability of the CSRR used in the prediction by computing the ratio of the query's atoms that match the substructures in the CSRR (yellow highlighting). The confidence score is calculated by adding the substrate-query, product-prediction, and atom efficiency values. The two examples show the components of the confidence score for two structurally related queries that produce different confidence scores due to greater difference in atom efficiency between the molecules.

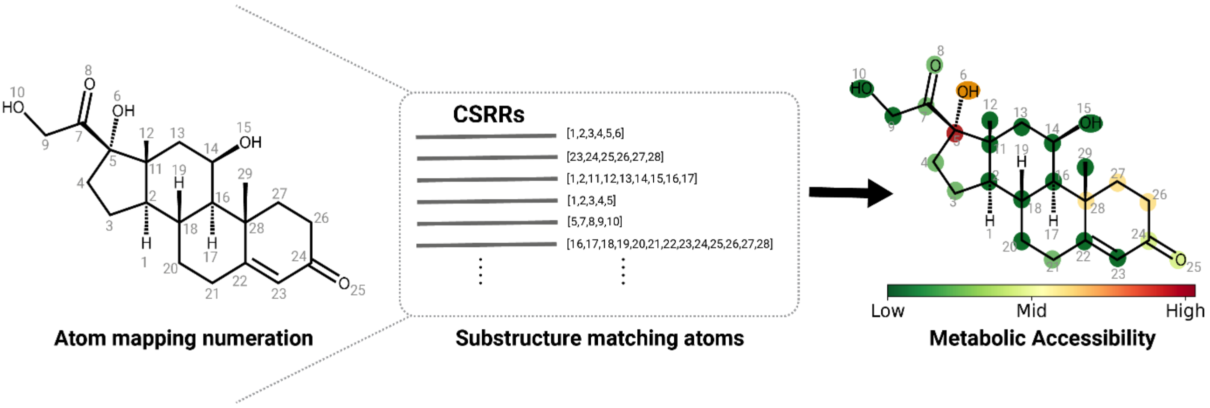

Figure S4. Implementation of metabolic accessibility in MicrobeRX. For each prediction, MicrobeRX assesses the number of atoms that match between the CSRR and the query (Fig. S2). As a result, metabolic accessibility is defined as the frequency with which each atom is recognized in the CSRRs, which is represented by a color scale. In the case of hydrocortisone (DB00741), the atom pair C-OH, which corresponds to atoms 5 and 6, appears most frequently in all of the CSRRs used to predict this drug's metabolites. As a result, the hydroxyl group is highlighted as being more metabolically accessible than the other hydrocortisone atoms.

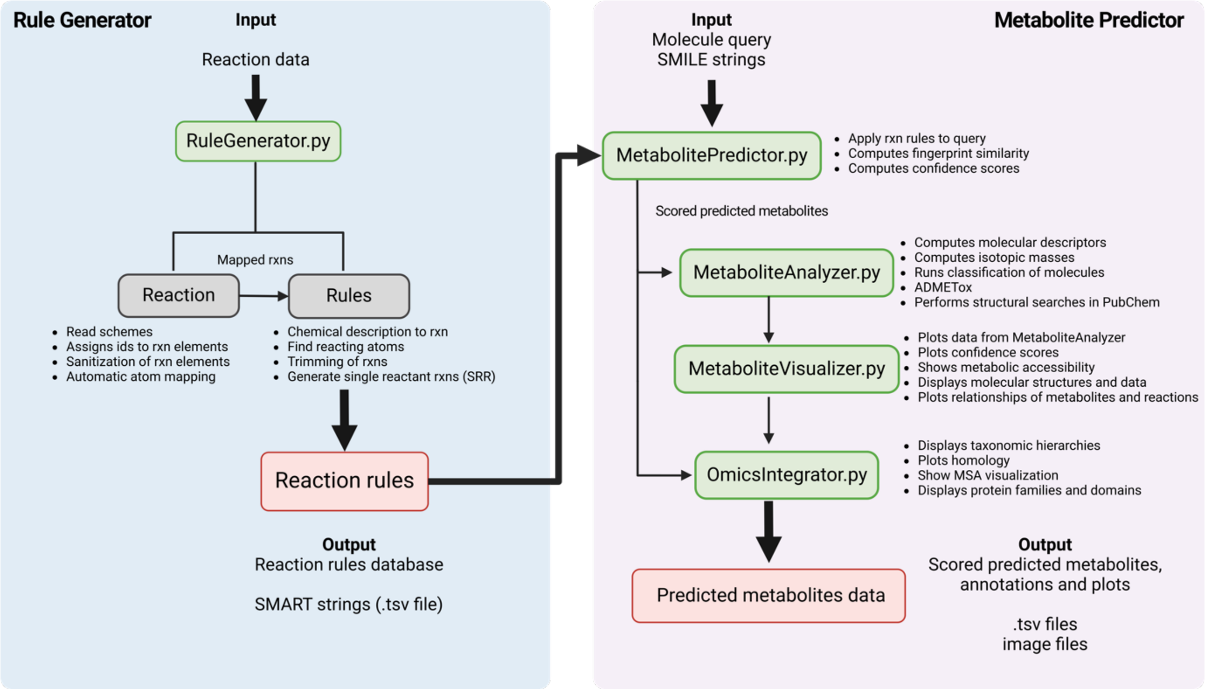

Figure S5. Schematic representation of MicrobeRX Python library. Overview of the MicrobeRX Python library illustrating the integration of reaction data from GEMs of human and gut microbiomes and the various modules within MicrobeRX, such as *RuleGenerator* and *MetabolitePredictor* (green boxes).

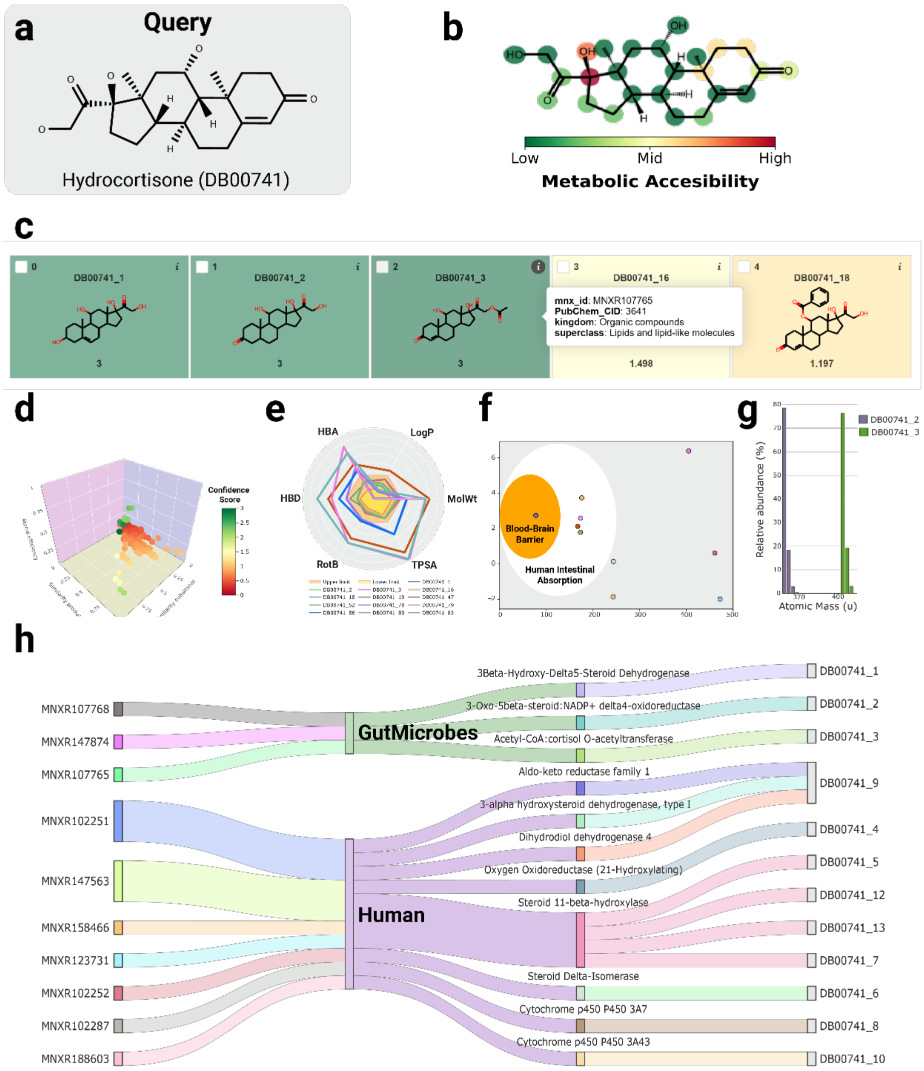

Figure S6. Example of analysis and visualization outputs from the *MetaboliteAnalyzer* and *MetaboliteVisualizer* modules for hydrocortisone. (a) Molecular structure of the query for the prediction (usually SMILES). **(b)** Per atom metabolic accessibility diagram. **(c)** Five top predicted metabolites, sorted by confidence score and including PubChem annotation and structural classification. **(d)** 3D scatter plot for the different component's confidence scores (x: substrate similarity, y: product similarity, z: atom efficiency). **(e)** Lipinski's rule of five bioavailability radar plot based in molecular descriptors (MolWt: molecular weight, LogP: octanol-water partition coefficient, TPSA: Topological Polar Surface Area, RotB: number of rotatable bonds, HBD: number of hydrogen bond donors, HBA: number of hydrogen bond acceptors). **(f)** ADMETox (BOILED-Egg) plot including the limits for Blood Brain Barrer and Human Intestinal Absorption accessibilities. **(g)** Bar plot of isotopic mass decomposition for two predicted metabolites. **(h)** Sankey diagram depicting relationships between the reaction, origin, enzyme name, and metabolite produced.

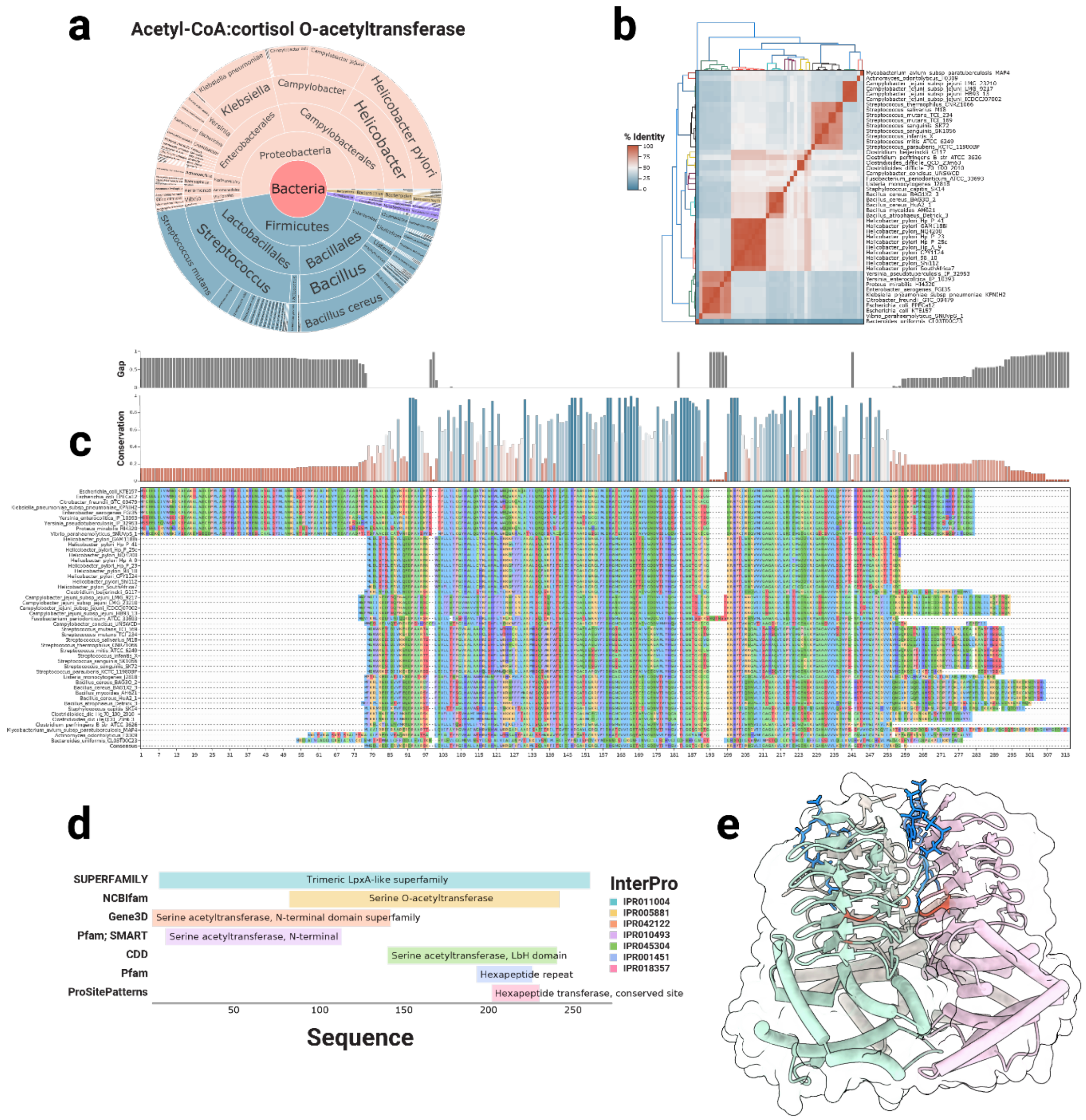

Figure S7. Example of outputs from the *OmicsIntegrator* module. (a) Sunburst plot of gut microbiome organisms per reaction/enzyme. **(b)** Cluster map of pairwise protein sequence comparisons for homology analysis. **(c)** Multiple sequence alignment with the consensus sequence (protein logo) highlighting conserved regions (blue bars). **(d)** Protein family search results from InterPro from a selected sequence of acetyl-CoA: cortisol o-acetyltransferase from gut microbes showing the distribution of protein domains. **(e)** Crystal structure of acetyl-CoA: cortisol acetyltransferase from *Salmonella typhimurium* (PBD: 8I06) complexed with CoA (blue), shown as a representative of the protein responsible for the metabolism of hydrocortisone.

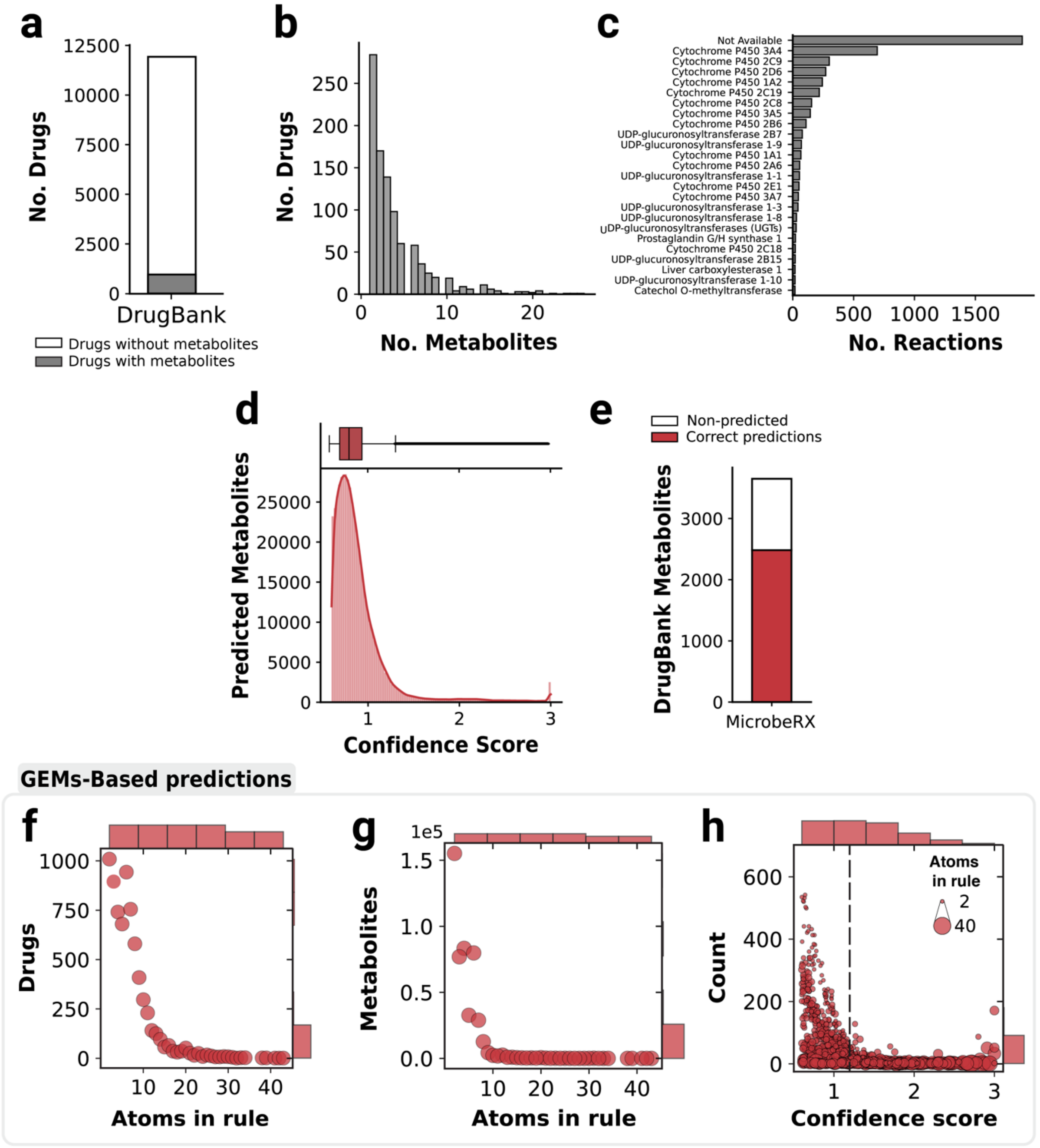

Figure S8. DrugBank metabolite dataset for MicrobeRX benchmark and predictions. (a) DrugBank v.5.1.12 contains 11,928 drug structures, of which only 954 drugs have reported metabolites**. (b)** Number of metabolites per drug reported in the DrugBank metabolites dataset. **(c)** Top 25 reported enzymes for the 3,650 metabolic reactions from the DrugBank database. **(d)** Confidence score distribution of the 480,458 MicrobeRX-predicted metabolites from 19,560 CSRRs derived from the 3,650 DrugBank reactions. Predictions with a confidence score above the upper whisker threshold (Q3 + 1.5*IQR ≈ 1.206) are considered high-confidence. **(e)** Proportion of correctly predicted metabolites from the whole DrugBank metabolites dataset. **(f)** Number of drugs processed depending on the number of atoms in the GEM-based CSRRs. **(g)** Number of metabolites predicted by the GEM-based CSRRs. **(h)** Confidence score values depending on the number of atoms used in the GEM-based CSRR for the predictions. Black dashed line indicates the selection confidence threshold of 1.2.

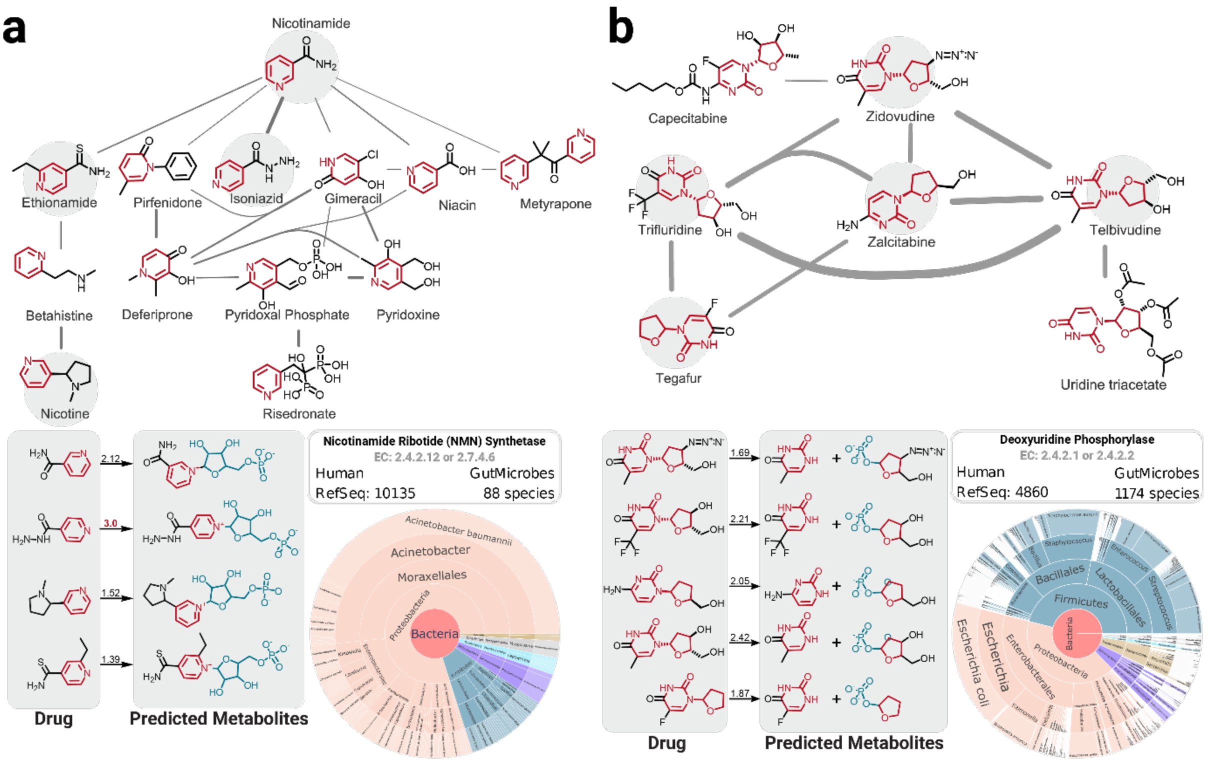

Figure S9. Predicted microbial metabolism of pyrimidine-containing drugs including nicotine and 5-fluorouracil (5-FU) by human and gut microbes. (a) Top panel displays the Molecular Similarity Network (MSN) of pyrimidine drugs (including nicotine) biotransformed by Nicotinamide Ribotide (NMN) Synthetase (NMNS, EC: 2.4.2.12). Edge size is the similarity between molecules at a 40% cutoff for constructing the tree. **(b)** Top panel displays the MSN of pyrimidine drugs (including tegafur) biotransformed by the periplasmic Deoxyuridine Phosphorylase (DURIPP, EC: 2.4.2.1). Edge size is the similarity between molecules at a 75% cutoff for constructing the tree. Lower panels represent the predicted metabolites from drugs selected in the MSNs (gray circles in **a** and **b**). The confidence score for each prediction is shown above the reaction arrow. Cofactors and secondary products are not shown for simplicity. The RefSeq identifier for human and the number of bacterial species containing the reaction are shown as a sunburst plot of enzyme phyla distribution. The maximum common substructure of the drugs is shown in red. Molecular substructures in blue are the predicted modifications from MicrobeRX.

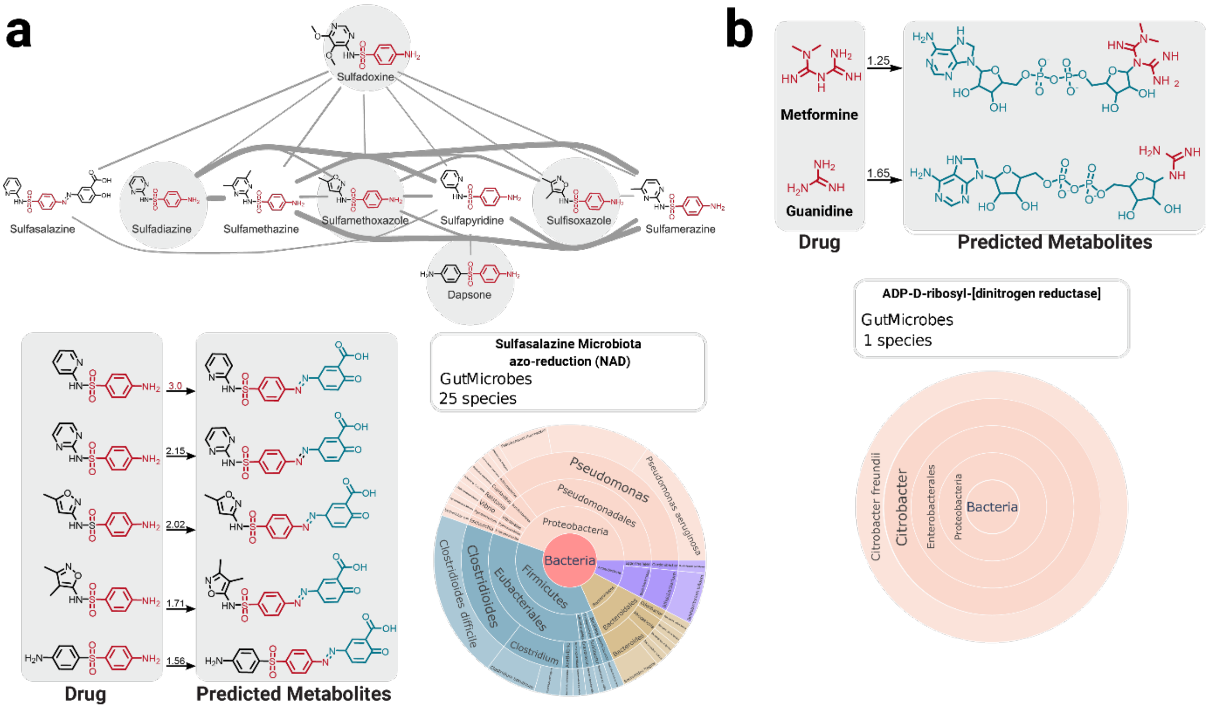

Figure S10. Example of microbial drug metabolism. (a) MSN of sulfonamides metabolized by the sulfasalazine microbial azo-reduction (SSZ_AR_NADi). Edge size is the similarity between molecules at a 70% cutoff for constructing the tree. **(b)** Metformin metabolism by *Citrobacter freundii*, producing ADP-D-ribose-metformin. The figure includes the chemical structures of the drugs and their metabolites, with confidence scores for each prediction shown above the reaction arrows. Cofactors and secondary products are not shown for simplicity. The number of bacterial species containing the relevant enzymes is shown as a sunburst plot of enzyme phyla distribution. Maximum common substructures of the drugs are shown in red. Molecular substructures in blue are the predicted modifications from MicrobeRX.

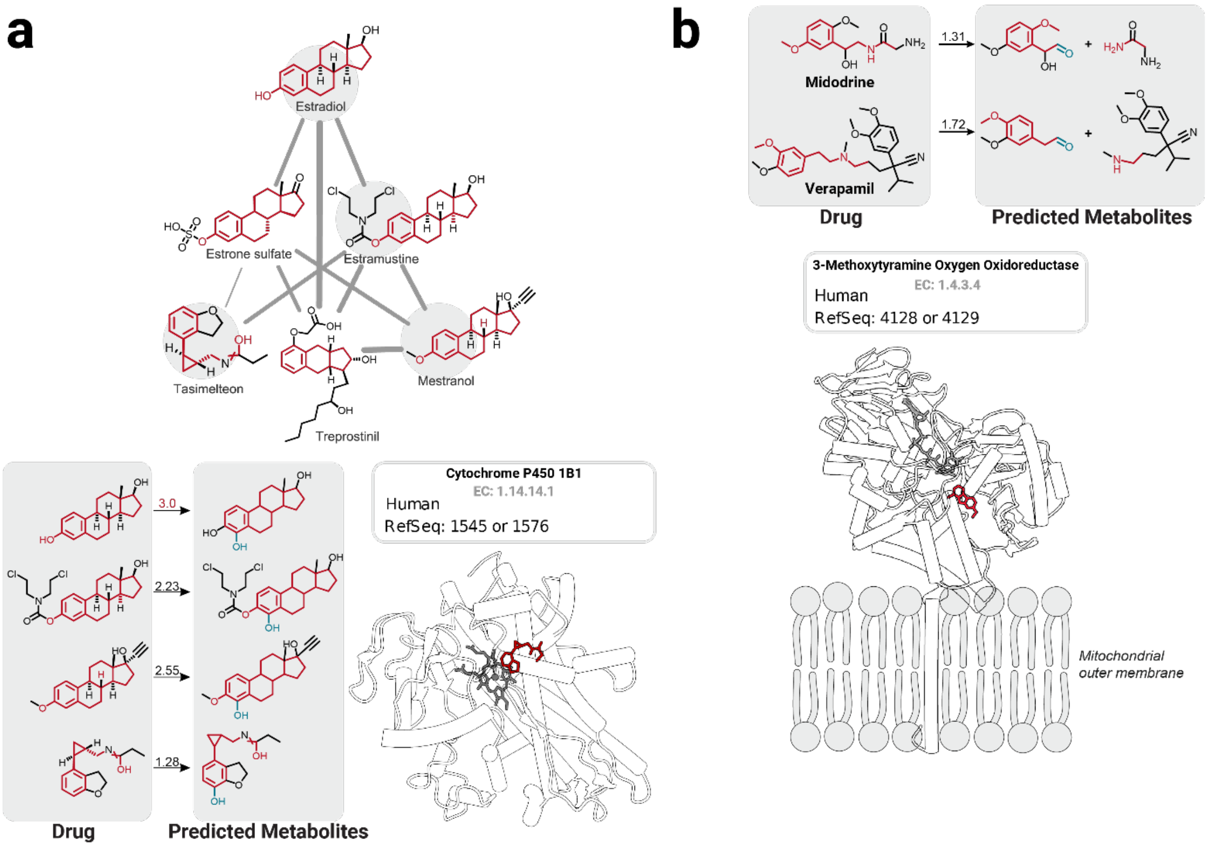

Figure S11. Examples of predicted human metabolism. (a) MSN of estrane-derivatives, including estradiol, by human CYP450 1B1 (EC: 1.14.14.1). Edge size is the similarity between molecules at a 50% cutoff for constructing the tree. **(b)** Predicted biotransformation of midodrine and verapamil by human 3-Methoxytyramine: Oxygen Reductase (3MOXTYROX, EC: 1.4.3.4). Confidence scores for each prediction are shown above the reaction arrows. Cofactors and secondary products are not shown for simplicity. For each enzyme, the lower panel includes the best molecular docking predicted pose of tasimelteon (red sticks, -9.6 kcal/mol) in the catalytic site of CYP450 1B1 (PDB: 6IQ5) and midodrine (red sticks, -7.5 kcal/mol) in 3MOXTYROX (PDB: 2Z5Y). Maximum common substructure of the drugs is shown in red. Molecular substructures in blue are the predicted modifications from MicrobeRX.

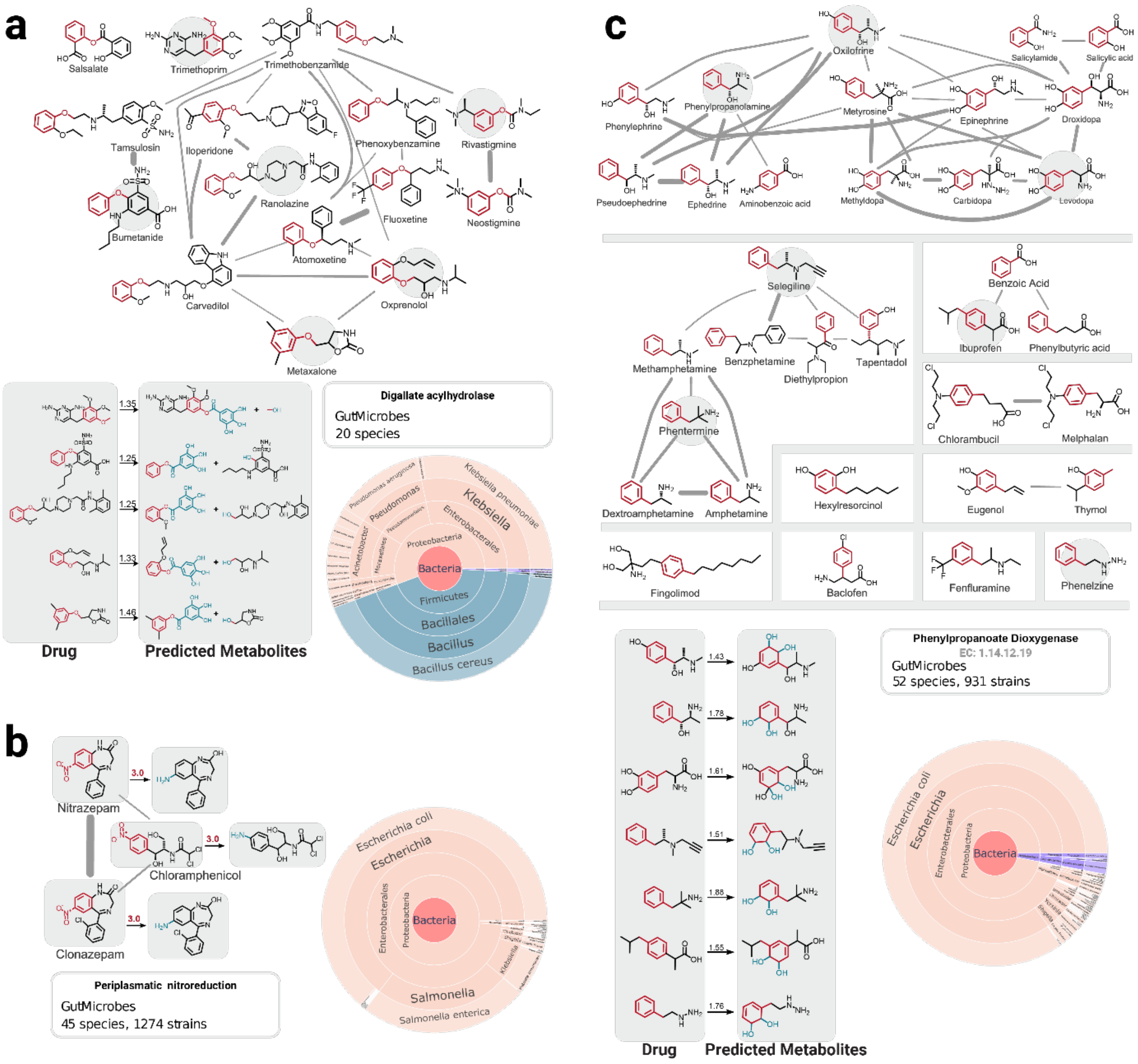

Figure S12. Examples of chemo- and taxa-specific microbial metabolism. (a) Metabolism of drugs containing anisole groups by digallate acylhydrolase (DGLTAH) in Proteobacteria and Firmicutes. Edge size is the similarity between molecules at a 50% cutoff for constructing the tree. **(b)** Metabolism of nitroaromatic drugs by periplasmatic nitroreductase (NRepp) in Proteobacteria, highlighting the prevalence of this enzyme in specific bacterial strains. **(c)** Biotransformation of benzene-containing drugs by phenylpropanoate dioxygenase (PPPNDO, EC: 1.14.12.19) in pathogens such as *Escherichia coli* and *Shigella flexneri*, with detailed structural changes during the metabolic process. Edge size is the similarity between molecules at a 55% cutoff for constructing the tree. Confidence scores for each prediction are shown above the reaction arrows. Cofactors and secondary products are not shown for simplicity. The number of bacterial species containing the relevant enzymes is shown as sunburst plots of enzyme phyla distribution. Maximum common substructures of the drugs are shown in red. Molecular substructures in blue are the predicted modifications from MicrobeRX.

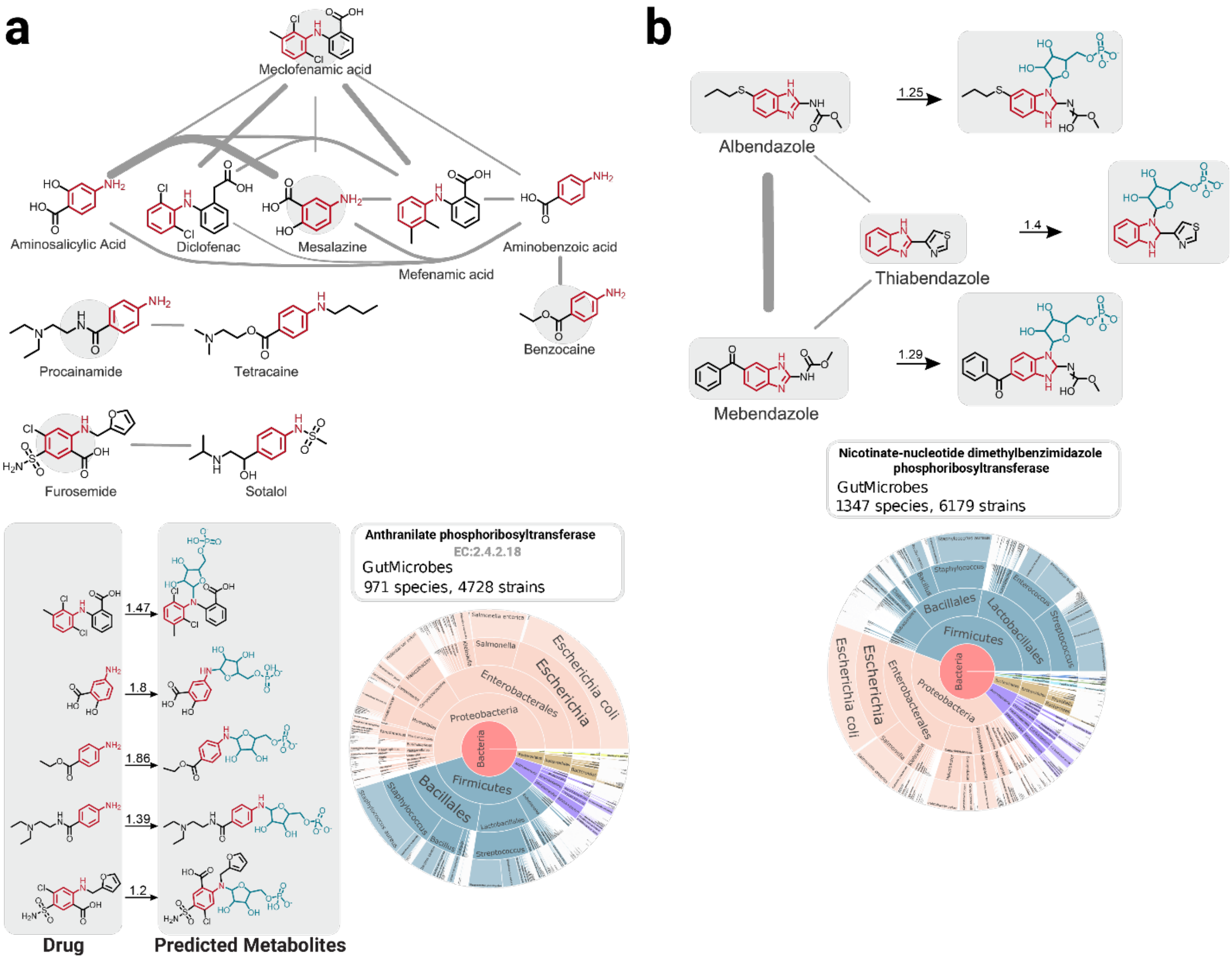

Figure S13. Examples of extended metabolism by gut microbiota. (a) Metabolism of endogenous-like compounds, including benzocaine by microbial Anthranilate phosphoribosyltransferase (ANPRT, EC: 2.4.2.18). Edge size is the similarity between molecules at 50% cutoff for constructing the tree. **(b)** Predicted biotransformation of benzimidazole antiparasitic by the Nicotinate-nucleotide dimethylbenzimidazole phosphoribosyltransferase (NNDMBRT) from the NAD metabolism pathway, showing the potential formation of α-ribazole-phosphate forms. Edge size is the similarity between molecules at 40% cutoff for constructing the tree. The figure includes chemical structures, with confidence scores for each prediction shown above the reaction arrows. Cofactors and secondary products are not shown for simplicity. The number of bacterial species containing the relevant enzymes is shown as sunburst plots of enzyme phyla distribution. Maximum common substructures of the drugs are shown in red. Molecular substructures in blue are the predicted modifications from MicrobeRX.
